# Putrescine Is an Intraspecies and Interkingdom Cell-Cell Communication Signal Modulating the Virulence of *Dickeya zeae*

**DOI:** 10.3389/fmicb.2019.01950

**Published:** 2019-08-21

**Authors:** Zurong Shi, Qingwei Wang, Yasheng Li, Zhibing Liang, Linghui Xu, Jianuan Zhou, Zining Cui, Lian-Hui Zhang

**Affiliations:** ^1^Guangdong Province Key Laboratory of Microbial Signals and Disease Control, South China Agricultural University, Guangzhou, China; ^2^Integrative Microbiology Research Centre, South China Agricultural University, Guangzhou, China; ^3^College of Agriculture and Biology, Zhongkai University of Agricluture and Engineering, Guangzhou, China

**Keywords:** putrescine, cell-cell signaling, pathogen-host communication, polyamine, regulatory elements, regulatory network

## Abstract

The infections caused by *Dickeya zeae* become a severe problem in recent years, but the regulatory mechanisms that govern the bacterial virulence remain to be fragmental. Here we report the investigation of potential involvement of polyamines in regulation of *D. zeae* virulence. We showed that null mutation of *speA* encoding arginine decarboxylase dramatically decreased the bacterial swimming motility, swarming motility and biofilm formation, and exogenous addition of putrescine effectively rescues the defective phenotypes of *D. zeae*. HPLC and mass spectrometry analysis validated that *speA* was essential for production of putrescine in *D. zeae*. In addition, we demonstrated that *D. zeae* EC1 could detect and response to putrescine molecules produced by itself or from host plant through specific transporters. Among the two transporters identified, the one represented by PotF played a dominated role over the other represented by PlaP in modulation of putrescine-dependent biological functions. Furthermore, we provided evidence that putrescine signal is critical for *D. zeae* EC1 bacterial invasion and virulence against rice seeds. Our data depict a novel function of putrescine signal in pathogen-host communication and in modulation of the virulence of an important plant bacterial pathogen.

## Introduction

*Dickeya zeae*, the causative agents of maize stalk rot and rice foot rot diseases, is previously named as *Erwinia chrysanthemi* pv. *zeae* (Sinha and Prasad, 1977; Goto, 1979; Nassar et al., 1994; Hussain et al., 2008). More recently, *D. zeae* was also shown to cause severe bacterial soft rot disease in banana, which is an important economical crop in many tropical and subtropical countries and regions (Zhang et al., 2013). *D. zeae* is much less studied compared with other well characterized species of the *Dickeya* genus, such as *D. dadantii*, formally known as *Erwinia chrysanthemi*, which are known to deploy a range of virulence determinants to enhance its competitive advantages against its host (Barras et al., 1994; Collmer and Bauer, 1994). In recent years, we found that *D. zeae* strain EC1 produces two polyketide phytotoxins, known as zeamine and zeamine II, which are able to inhibit rice seed germination (Zhou et al., 2011; Cheng et al., 2013). Deletion of the genes encoding zeamine and zeamine II production abrogated and attenuated the bacterial virulence, respectively (Zhou et al., 2011; Cheng et al., 2013), suggesting that zeamines are the key virulence determinants of *D. zeae*. In addition, zeamines are also potent antibiotics, which effectively kill a wide range of bacterial and fungal pathogens at low end of micromolar concentrations (Wu et al., 2010; Liao et al., 2014). In addition to phytotoxin zeamines, evidence suggests that other virulence associated traits might also contribute to the virulence of *D. zeae*. The acyl-homoserine lactone (AHL) quorum sensing (QS) system is widely conserved in Gram-negative bacteria, including *D. zeae.* The AHL QS system is consist of a *luxI* homolog, which encodes for AHL signal biosynthesis, and a *luxR* homolog, which encodes an AHL-dependent transcription factor. *D. zeae* has only one set of AHL QS system with *expI*, the homolog of *luxI*, encoding for biosynthesis of the QS signal *N*-(3-oxo-hexanoyl)-homoserine lactone (OHHL) (Hussain et al., 2008). Disruption of *expI* had little effect on zeamines production in a rich medium, but resulted in increased bacterial cell motility, decreased production of extracellular enzymes, and partially attenuated bacterial virulence against dicotyledonous plant Chinese cabbage and monocotyledonous plant rice (Hussain et al., 2008).

Evidence suggests that the AHL QS system may regulate the virulence genes through the transcription factor SlyA. Genetic analysis showed that SlyA positively regulates biofilm formation, virulence on rice seeds and negatively controls bacterial motility, and *in trans* expression of *slyA* in the *expI* mutant restored the phenotypes of bacterial motility and biofilm formation (Zhou et al., 2016), suggesting that SlyA may act at the downstream of the AHL QS system. In contrast to the AHL QS system that has only a minor effect on zeamines biosynthesis, null mutation of *slyA* led to a significant reduction in zeamines production (Zhou et al., 2016). The findings suggest that the AHL system may only partially influence the biological functions of SlyA, indicating the complexity of virulence regulatory mechanisms in *D. zeae*.

Spermidine, spermine, and putrescine constitute a group of ubiquitous aliphatic small polycationic molecules known as polyamines, which are widely distributed from bacteria to plants and animals. The evidence of polyamine molecules as signaling molecules in microorganisms has been accumulating in recent years. We reported previously that spermidine and spermine from mammalian host are the interkingdom cell-cell communication signals, which are taken up through a potent ABC transporter SpuBCDEF and specifically induce the transcriptional expression of the genes encoding the type III secretion system in a human bacterial pathogen *Pseudomonas aeruginosa* (Zhou et al., 2007; Wu et al., 2012). In another human pathogen *Proteus mirabilis*, it is putrescine that acts as an extracellular signal required for bacterial swarming motility and invasion ability (Sturgill and Rather, 2004; Kurihara et al., 2013). Putrescine has also been known for its role in modulation of biofilm formation and disassembly by human pathogen *Yersinia pestis* and environmental bacterium *Shewanella oneidensis*, respectively (Patel et al., 2006; Ding et al., 2014). Additionally, evidence indicates that putrescine produced by the plant pathogen *Ralstonia solanacearum* might also act on host to accelerate plant wilt disease (Lowe-Power et al., 2018). Putrescine biosynthesis in bacteria has two alternative pathways, one pathway starts with ornithine by ornithine decarboxylase (SpeC) known as ODC pathway (Boyle et al., 1984). The second pathway involves the sequential conversion from arginine by arginine decarboxylase (SpeA) and agmatinase (SpeB) to putrescine (Boyle et al., 1984). The triamine spermidine is formed from putrescine by addition an aminopropyl group derived from decarboxylated *S*-adenosylmethionine by the spermidine synthase encoded by *speE* (Tabor et al., 1986; Tabor and Tabor, 1987).

To test whether polyamines could also play a role in regulation of *D. zeae* virulence, we conducted a systemic deletion analysis on the genes encoding polyamine biosynthesis and transport. Our results showed that deletion of the arginine decarboxylase gene *speA* for putrescine biosynthesis could drastically change the patterns of bacterial motility and biofilm formation. We also found two putrescine specific transporters that collectively play a critical role for bacterial response to putrescine signals produced by bacteria or host plant in regulation of *D. zeae* motility and virulence. To our knowledge, this is the first report on modulation of bacterial motility by putrescine signals from eukaryotic cells.

## Materials and Methods

### Bacterial Strains, Plasmids, and Reagents

The bacterial strains and plasmids used in this study are listed in Supplementary Table S1. *D. zeae* EC1 and its derivatives were grown at 28°C in LB medium unless otherwise stated. Minimal medium (K_2_HPO_4_ 10.5 g/L, KH_2_PO_4_ 4.5 g/L, (NH)_2_SO_4_ 2 g/L, MgSO_4_.7H_2_O 0.2 g/L, FeSO_4_ 0.005 g/L, CaCl_2_ 0.01 g/L, MnCL_2_ 0.002 g/L, glycerol 2 g/L, and mannitol 2 g/L) was used for comparison of bacterial growth rate. Putrescine, spermidine, and spermine were purchased from Sigma-Aldrich. Antibiotics were added at the following final concentrations when required: kanamycin (Km), 100 μg/mL; streptomycin (Str), 50 μg/mL; gentamycin (Gm), 50 μg/mL; and tetracycline (Tc), 10 μg/mL.

### Generation of In-Frame Deletion Mutants and Complementation

The in-frame deletion mutants of *speA, speC, speE, potF*, and *plaP* were generated through homologous recombination as previously described (Zhou et al., 2011; Cheng et al., 2013), and named as Δ*speA*, Δ*speC*, Δ*speE*, Δ*potF*, and Δ*plaP*, respectively. The *speA/potF* and *speA/plaP* double deletion mutants were generated by deleting *potF* and *plaP* in the genetic background of the *speA* deletion mutant and designated as Δ*speA*Δ*potF* and Δ*speA*Δ*plaP*, respectively. The *speA/potF/plaP* triple gene deletion mutant Δ*speA*Δ*potF*Δ*plaP* was generated by deleting *plaP* using the double mutant Δ*speA*Δ*potF* as the parental strain. The detailed descriptions of strains and mutants were provided in Supplementary Table S1. All the primers used in this study were listed in Supplementary Table S2. Fragments containing about 500 bps upstream and downstream regions of target genes were amplified from the chromosomal DNA sample of *D. zeae* strain EC1, respectively, which were linked by second round PCR with the forward primer of the upstream fragment and the reverse primer of the downstream fragment. The fusion PCR fragments were digested with restriction enzymes and ligated to the vector pKNG101 digested with the same enzymes. The resultant constructs were transformed into *Escherichia coli* CC118. *D. zeae* mutants were generated by tri-parental mating following the protocol described previously (Cheng et al., 2013).

For complementation analysis, the encoding regions of genes *speA*, *potF*, and *plaP* were amplified by the primers listed in Supplementary Table S2. The PCR products were digested with restriction enzymes and then cloned into the expression vector pBBR1-MCS4 digested with the same enzymes. The complementation constructs were introduced into corresponding mutants by tri-parental mating and confirmed by PCR analysis.

### Bacterial Motility and Biofilm Assays

To examine changes in bacterial swimming motility, 2 μL overnight cultures of *D. zeae* strains were spotted inside the agar at the center of semisolid Bacto tryptone agar medium (per liter contains 10 g bacteriological peptone, 5 g NaCl, 3 g agar). After incubation for 24 h at 28^o^C, the diameters of swimming motility were measured. The swarming motility was assayed under the same conditions, except that bacterial cells were spotted onto the semisolid medium containing (per liter) 5 g tryptone, 5 g NaCl, and 4 g agarose. The experiments were repeated three times in triplicates.

For measurement of biofilm formation, 1 μL overnight cultures of *D. zeae* strain EC1 and its derivatives were added to a 96-well plate containing 99 μL fresh SOBG medium (tryptone, 20 g/L, yeast extract, 5 g/L, MgSO4.7H_2_O 2.4 g/L, NaCl 0.5 g/L, KCl 0.186 g/L, glycerol 20 mL/L). The plates were incubated at 28 ^o^C with shaking at 150 rpm for 18 h and the liquid cultures were removed. The attached bacterial cells were incubated with 0.1% crystal violet (CV) for 15 min before draining the liquid and washing three times with water. CV molecules retained by attached bacterial cells were solubilized with 95% ethanol. The amount of dye bound, representing the mass of attached bacterial cells, was monitored by measuring the absorbance at 570 nm on a microplate spectrophotometer. The experiments were repeated three times with triplicates.

### Chemical Complementation With Exogenous Polyamines

Different concentrations of putrescine and spermidine were added to semisolid medium as indicated for determination of their activity in restoration of swimming motility phenotype of the mutant Δ*speA*. Swimming motility of the *D. zeae* strains were quantified using the method described above. To rescue the swarming motility and biofilm formation phenotypes of the mutant Δ*speA*, putrescine and spermidine were added to corresponding media at a final concentration of 0.1 mM, respectively. The experiments were repeated three times with triplicates.

### Bacterial Sample Preparation and Polyamine Derivatization

The cellular levels of putrescine in wild type strain EC1 and mutant Δ*speA* cells were measured based on the methods described previously with minor modifications (Kashiwagi and Igarashi, 1988; Kurihara et al., 2008; Lee et al., 2009; Sethi et al., 2011). Briefly, bacterial cells were grown to an OD_600_ of 1.7 at 28°C with shaking at 200 rpm in minimal medium, then pelleted and the wet weight of bacterial cells was recorded. Cell pellets were washed with ddH_2_O for three times and resuspended with 600 μL lysis buffer (20 mM MOPS, pH 8.0, 10 mM NaCl, 4 mM MgCl_2_), then lysed by three cycles of freeze/thawing with liquid nitrogen. Trichloroacetic acid (40%) was added to the lysed cell suspension at a final concentration of 10%, and the mixtures were incubated on ice for 30 min. The supernatants were collected by centrifugation and used for derivatization. For each sample (500 μL), derivatization was carried out by adding 125 μL of 2 M NaOH and 100 μL of benzoyl chloride and incubated for 30 min at room temperature with constant shaking (Sethi et al., 2011). Benzoylated putrescine was used for analysis with high performance liquid chromatography mass spectrometry (LC-MS). The derivatized samples were separated by using an Eclipse Plus C18 (Aglent) column fitted with a 100- by 2.1 mm guard column with a flow rate of 0.3 mL/min. Mass spectroscopy (Aglent 6540B Q-TOF) was used to verify the identity of each peak observed in HPLC fractions. The experiments were repeated three times in triplicates. A standard curve was generated by using various concentrations of benzoylated putrescine in duplicates.

### Culture With Rice Seedling Extract

Seeds of rice variety CO39 were soaked with water at room temperature for 24 h, transferred onto moistened gauze in a plastic sorting box and were incubated at 28°C with a 16 h light and 8 h dark cycle for 3 days. About 3 g seedlings were placed in mortar and grinded with liquid nitrogen. The powders of seedlings were homogenated in 30 mL ddH_2_O and stored at 4°C for 24 h to extract free polyamines, then centrifuged at 8,000 rpm for 15 min and supernatants were collected. The sterilized supernatants were mixed with the semi-preparative swimming medium at the ratio of 1:5. The semi-preparative swimming medium was added with the same proportion of sterilized water as blank control. Swimming motility was measured as described above. The rice seedling extracts were also taken for polyamines analysis with LC-MS as described above. The experiments were repeated three times in triplicates.

### Transcriptome and RT-PCR Analyses

Overnight cultures of strain EC1 and Δ*speA* were cultured in swimming motility medium with putrescine at the final concentration of 0.1 mM or without at 28°C to OD_600_ = 0.6. The RNA samples were prepared using the SV total RNA isolated system kit (Promega) and further purified using the RNA clean kit (Qiagen). The total RNA samples were treated with DNase I for degrading DNA. The mRNA samples were purified from total RNA using poly-T oligo-attached magnetic beads and fragmented into short fragments by mixing with fragmentation buffer. Then cDNA fragments were synthesized using random hexamer-primers, dNTPs, RNase H, and DNA polymerase I. The ends of the purified double-strand cDNA were repaired by adding adenine before purification of cDNA fragments with a QIAQuick Kit (QIAGEN, Germany). Sequencing adaptors were ligated to the cDNA fragments and PCR amplification was then performed to enrich the fragments. Finally, the library of cDNAs was constructed and sequenced on an Illumina Hisq platform. Sequenced Reads were obtained by base calling using CASAVA software and saved in FASTQ format. Reads were mapped to the whole genome sequence of *D. zeae* EC1 (CP006929.1). The gene expression level was calculated by using RPKM (Reads per Kilobase of Transcript per Million Reads Mapped) method (Wagner et al., 2012) and analyzed using DEseq (Wang et al., 2010). The genes with statistically significant changes in expression (| log2Ratio| ≥ 1 and *q* ≤ 0.05) were randomly selected for verification. Reverse transcription-polymerase chain reaction (RT-PCR) was performed by using StarScript II first-strand cDNA synthesis Mix following the manufacturer’s protocol (GenStar) with the RT-PCR primers listed in the Supplementary Table S2. The raw data were available under SRA accession number PRJNA516290.

### Rice Seed Germination and Bacterial Invasion Assays

The rice seed germination assay was conducted as previously described (Hussain et al., 2008). Briefly, overnight bacterial cultures were diluted in 10-fold series, and the CFU of each dilution was determined using heterotrophic plate counting assay. Thirty seeds of rice variety CO39 were added to 9 mL of bacterial dilution and incubated at room temperature for 5 h. The rice seeds were then washed three times with sterilized water and transferred onto two moistened filter papers in a petri dish. The seeds were then incubated at 28°C with a 16 h light and 8 h dark cycle, and sterilized water was added when necessary. Rice seeds were incubated with same amount of sterilized water as a blank control. The rate of seed germination was determined 5 days after treatment. The experiment was repeated four times.

For analysis of bacterial invasion, the encoding region of *gfp* was amplified by PCR, and cloned under the control of the *lac* promoter in the expression vector pLAFR3, which carries a tetracycline resistance gene. The resultant construct was introduced into *D. zeae* strain EC1 and its derivatives via tri-parental mating. Overnight cultures of GFP-labeled strains EC1, Δ*speA* and Δ*speA*Δ*potF*Δ*plaP* were resuspended and diluted with ddH_2_O to the CFU of 10^2^. Sixteen seeds of rice variety CO39 were added to 1 mL of bacterial dilution and incubated at room temperature for 5 h, and then transferred onto moistened filter papers in plates. Rice seeds were incubated with same amount of sterilized water as a blank control. The seeds were incubated at 28°C of 16 h light 8 h dark condition for 24 h. The husks of rice seeds were removed with one group containing 8 seeds per treatment being examined under a fluorescence microscope and the other containing same amount of seeds cut into small pieces and resuspended with 1 mL sterilized water. Samples were diluted accordingly and spread onto LB plates containing tetracycline, which were cultured for 24 h to count bacterial colony forming unit (CFU). The experiments were repeated three times.

### Statistical Analysis

For analysis of statistical significance, the data were analyzed using GraphPad Prism’s *t*-test and *P* < 0.05 was considered significant for all experiments.

## Results

### Null Mutation of *speA* Impairs Cell Motility and Biofilm Formation

Four enzymes are known to be associated with the biosynthesis of polyamines in *E. coli*, including arginine decarboxylase (SpeA, NCBI accession YP006135334.1), ornithine decarboxylase (SpeC, NCBI accession BAI27252.1), agmatinase (SpeB, NCBI accession BAE77000.1) and spermidine synthase (SpeE, NCBI accession BAB96695.1) (Boyle et al., 1984; Tabor et al., 1986; Tabor and Tabor, 1987). Blast analyses showed that *D. zeae* strain EC1 contains homologs of *speA* (NCBI accession WP_016940691.1), *speC* (NCBI accession WP_016942161.1), and *speE* (NCBI accession WP_016942102.1) (Zhou et al., 2015). Sequence alignment analysis showed that *speA*, *speC* and *speE* share about 86, 72, and 79% similarity at the peptide level with their counterparts in *E. coli*, respectively.

**FIGURE 1:**
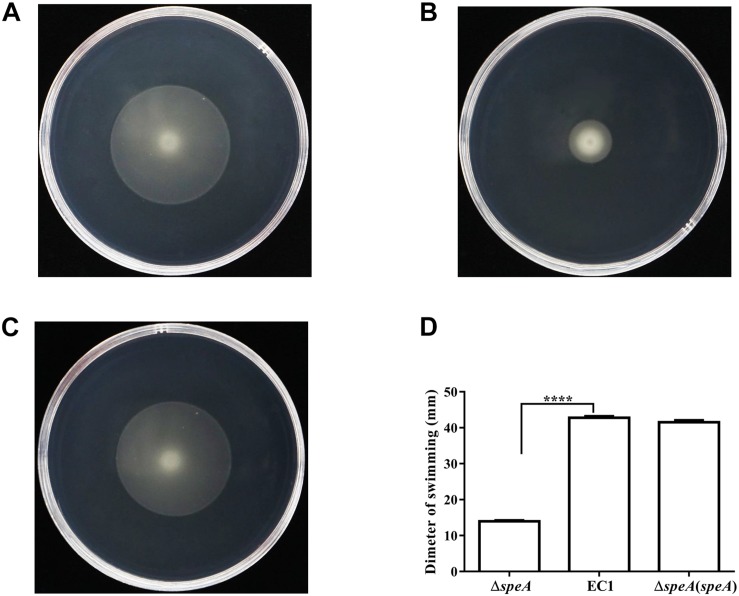
Mutation of the *speA* gene resulted in reduced swimming motility. **(A)** *D. zeae* wild type strain EC1, **(B)** the *speA*-disrupted mutant Δ*speA*, **(C)** strain Δ*speA*(*speA*) derived by *in trans* expressing *speA* in the mutant Δ*speA*. **(D)** Quantitative measurement of swimming motility of strain EC1 and its derivatives. The photographs were taken after incubation for 24 h at 28°C. The data shown are the means ± SE (*n* = 3). ^****^P < 0.0001.

**FIGURE 2:**
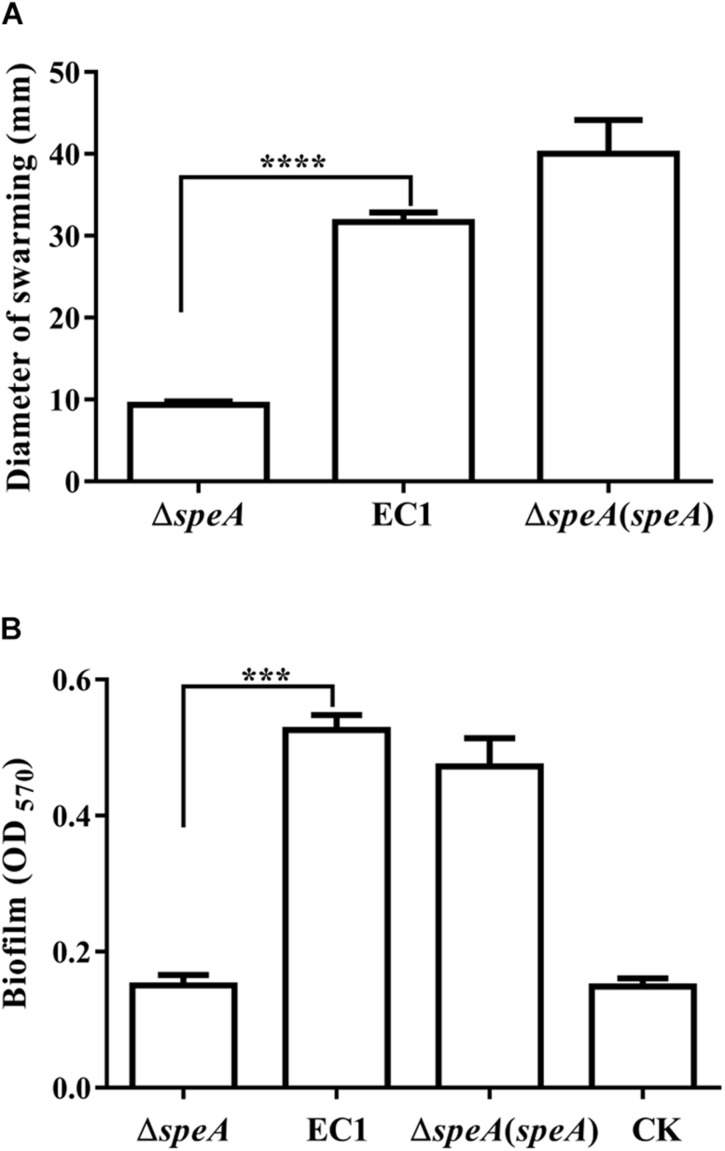
Disruption of *speA* decreased bacterial swarming motility and biofilm formation. **(A)** Swarming motility of strain EC1and its derivatives, the diameters were measured after incubation for 18 h at 28°C. **(B)** Biofilm formation of wild type and its derivatives. Bacterial strains were grown in SOBG medium at 28°C with shaking at 150 rpm for 18 h, and the medium without inoculation was used as a blank control (CK). The data shown are the means ± SE (*n* = 3). ^****^ *P* < 0.0001, ^∗∗∗^*P* < 0.001.

To determine the roles of polyamines in *D. zeae* physiology and pathogenesis, we constructed the in frame deletion mutants of *speA*, *speC*, and *speE* using *D. zeae* strain EC1 as a parental strain, and assayed for phenotype changes. Among these three mutants, the *speA* deletion mutant Δ*speA* showed the significantly decreased swimming motility (Figure 1), swarming motility and biofilm formation (Figures 2A,B), compared with the wild type strain EC1 and the complemented strain Δ*speA*(*speA*). However, deletion of *speC* did not cause any statistically significant phenotype changes, while abrogation of *speE* showed a marginal effect, if any, compared with the wild type strain EC1 (Supplementary Figure S1). Time course analysis found that the mutant Δ*speA* grew in a similar rate with the wild type strain EC1 and its complemented strain Δ*speA*(*speA*), indicating that the phenotypes of the *speA* mutant were not caused by reduced growth rate (Supplementary Figure S2). The above data thus suggest that the enzymatic product of SpeA might play a key role in modulation of cell motility and biofilm formation of *D. zeae*.

### Exogenous Addition of Putrescine Rescues Cell Motility and Biofilm Formation

**FIGURE 3:**
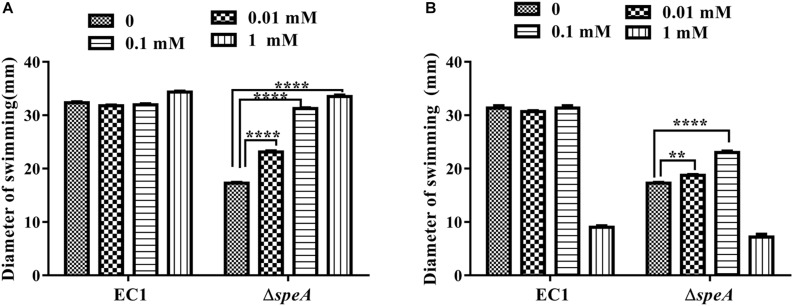
Effect of exogenous addition of polyamines on bacterial swimming motility. Bacterial strains were grown under the same condition but supplemented with different concentrations of putrescine **(A)** and spermidine **(B)**. The diameters of swimming motility were measured after incubation for 24 h at 28°C. The data shown are the means ± SE (*n* = 3). ^****^P < 0.0001, ^∗∗^*P* < 0.01.

**FIGURE 4:**
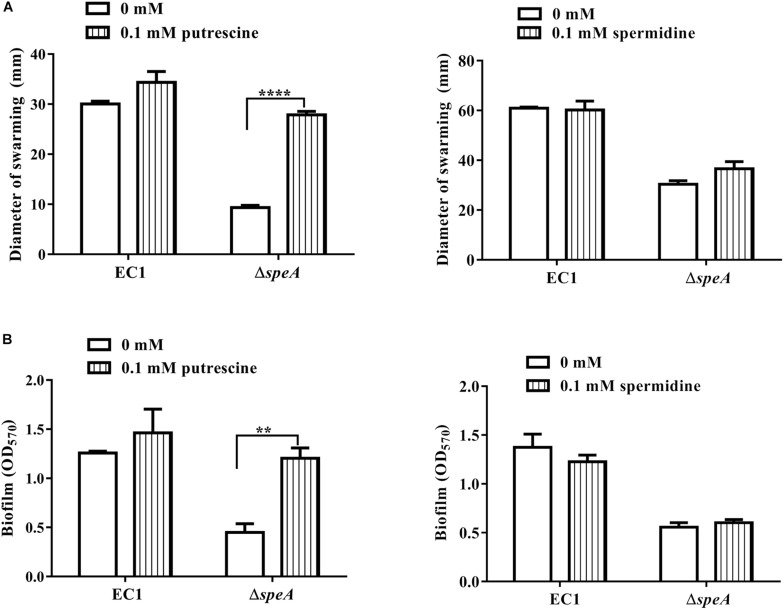
Effect of exogenous addition of putrescine or spermidine on swarming motility and biofilm formation. **(A)** Swarming motility of EC1 and its derivatives in the presence of 0.1 mM putrescine or spermidine, respectively, the diameter of warming zone was measured at 24 h after inoculation. **(B)** Effect of putrescine or spermidine on biofilm formation at a final concentration of 0.1 mM. The data shown are the means ± SE (*n* = 3). ^****^P < 0.0001, ^∗∗^*P* < 0.01.

Given that *speA* encodes the key enzyme of polyamine biosynthesis pathway in bacteria, and deletion of *speA* might decrease the intercellular levels of putrescine and spermidine, we set to determine whether exogenous addition of polyamine molecules could rescue the defective phenotypes of the mutant Δ*speA*. As shown in Figure 3, exogenous addition of putrescine could increase the swimming motility of the mutant Δ*speA* in a dosage dependent manner, whereas the effect of spermidine was marginal, and the molecule even inhibited the bacterial swimming motility at a high concentration of 1 mM. Similarly, addition of putrescine at a final concentration of 0.1 mM rescued the mutant phenotypes of swarming motility and biofilm formation (Figures 4A,B). Taken together, these data suggest that inactivation of *speA* might reduce the intracellular putrescine concentration, which led to decreased cell motility and reduced biofilm formation of *D. zeae*.

### Transporters Are Required for Response to Extracellular Putrescine

Previous studies showed that polyamines molecules are transported into cells through membrane bound transporters. In the well characterized *E. coli*, four putrescine specific transporters, i.e., PotE, PotFGHI, PuuP, and PlaP, and one spermidine/putrescine transporter PotABCD have been identified and characterized (Furuchi et al., 1991; Kashiwagi et al., 1992; Pistocchi et al., 1993; Kurihara et al., 2009, 2011). Analysis of the *D. zeae* genome unveiled two putrescine specific transporter loci annotated as *potFGHI* and *plaP*, respectively. Amino acid sequence comparison showed that PotF and PlaP from *D. zeae* EC1 share about 81% identity to the substrate binding proteins PotF and PlaP of *E. coli*, respectively. Therefore, the *potF* and *plaP* genes in *D. zeae* EC1 are highly likely to encode putrescine binding proteins of the cognate transporter systems. To determine their roles in putrescine transportation, *potF* and *plaP* were deleted in the wild type strain EC1 and the mutant Δ*speA* genetic background, respectively. The results showed that single deletion of *potF* and *plaP* or deletion of both *potF* and *plaP* did not affect bacterial swimming motility in comparison with the wild type strain EC1 (Supplementary Figure S3E). We therefore generated the triple-deletion mutant Δ*speA*Δ*potF*Δ*plaP* by deletion of *speA/potF/plaP* consecutively in the wild type strain *D. zeae* EC1. The results showed that in the swimming plate, the swimming zone of triple-deletion mutant Δ*speA*Δ*potF*Δ*plaP* became smaller than the single deletion mutant Δ*speA* (Supplementary Figures S3A,B). Interestingly, under the same conditions, while *in trans* expression of *potF* under the control of a constitutive promoter P*lac* in Δ*speA*Δ*potF*Δ*plaP* could restore the mutant phenotype (Supplementary Figure S3C), complementation of *plaP* under the control of same promoter in Δ*speA*Δ*potF*Δ*plaP* failed to rescue the mutant motility (Supplementary Figure S3D). This suggests that the transporter PlaP might not be functional when the bacterial cells grown in the swimming plate.

For verification, we generated the double-deletion mutants Δ*speA*Δ*plaP* and Δ*speA*Δ*potF*, respectively, and compared their swimming motility with the single-deletion mutant Δ*speA* and triple-deletion mutant Δ*speA*Δ*potF*Δ*plaP*. The results showed that the swimming motility of the double deletion mutant Δ*speA*Δ*potF* and triple-deletion mutant Δ*speA*Δ*potF*Δ*plaP* were decreased by about 33 and 35%, respectively, in comparison with the mutant Δ*speA* (Supplementary Figure S3G). However, consistent with the *plaP* complementation data (Supplementary Figure S3D), the other double deletion mutant Δ*speA*Δ*plaP* showed a similar swimming motility to its parental strain Δ*speA* (Supplementary Figure S3F).

**FIGURE 5:**
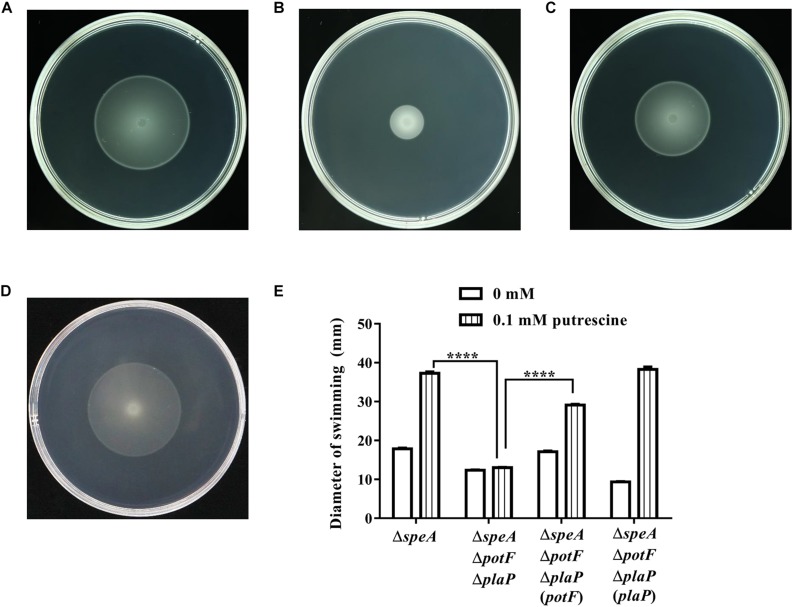
Influence of transporters on putrescine uptaking. The plate bioassay was performed using swimming motility supplemented with 0.1 mM of putrescine for 24 h at 28°C. **(A)** The *speA*-disrupted mutant; **(B)** The *potF-plaP* double disrupted mutant in the *speA*-disrupted genetic background (Δ*speA*Δ*potF*Δ*plaP*); **(C,D)** the complemented strains Δ*speA*Δ*potF*Δ*plaP*(*potF*) **(C)** and Δ*speA*Δ*potF*Δ*plaP*(*plaP*) **(D)**; **(E)** Quantitative measurement of swimming motility of Δ*speA* and its derivatives with or without putrescine (CK). The data shown are the means ± SE (*n* = 3). ^****^P < 0.0001.

Given that swimming plate might contain only a small amount of putrescine, likely from the medium component bacteriological peptone, we then tested the roles of transporters in the presence of exogenous putrescine. The results showed the swimming motility of the triple-deletion mutant Δ*speA*Δ*potF*Δ*plaP* was much reduced compared with the single deletion mutant Δ*speA* (Figures 5A,B). Similar to the complementation experiment without exogenous addition of putrescine (Supplementary Figure S3C), complementation of Δ*speA*Δ*potF*Δ*plaP* with *potF* largely restored its swimming motility (Figure 5C). Surprisingly, however, *in trans* expression of *plaP* in the mutant Δ*speA*Δ*potF*Δ*plaP* could fully rescue its swimming motility in a capacity better than *potF* (Figures 5C,D), which differs from the same complementation experiment but without exogenous addition of putrescine (Supplementary Figures S3C,D). Superior performance of PlaP transporter than PotF transporter was further confirmed when the bacterial motilities of *speA/potF* and *speA/plaP* double deletion mutants were compared in the presence of exogenous putrescine (Supplementary Figure S3F). Quantitative analysis showed that exogenous addition of putrescine significantly increased the swimming motility of the mutant Δ*speA* and the complemented strains Δ*speA*Δ*potF*Δ*plaP*(*potF*) and Δ*speA*Δ*potF*Δ*plaP*(*plaP*), whereas the same treatment had no effect on the motility of triple-deletion mutant Δ*speA*Δ*potF*Δ*plaP* (Figure 5E), validating the key role of two transporters in efflux of exogenous putrescine in modulation of bacterial motility.

PotF and PlaP from *D. zeae* share a high similarity with their counterparts in *E. coli* with over 81% identity at amino acid level. Among them, PotF was previously demonstrated as a specific putrescine-binding protein (Vassylyev et al., 1998), whereas PlaP is a relatively newly discovered putrescine importer in *E. coli* (Kurihara et al., 2011). For verification, we tested the role of transporter of PlaP in the presence of exogenous putrescine, spermidine and spermine at the final concentration of 0.1 mM, respectively. As expected, exogenous addition of putrescine significantly increased the swimming motility of the complemented strain Δ*speA*Δ*potF*Δ*plaP*(*plaP*), whereas the same treatment had no effect on the motility of triple-deletion mutant Δ*speA*Δ*potF*Δ*plaP*, validating the key role of PlaP in uptake of exogenous putrescine. However, exogenous addition of same concentration of spermidine or spermine had no effect on swimming motility of strain Δ*speA*Δ*potF*Δ*plaP*(*plaP*) (Supplementary Figure S4). These results are consistent with the data shown in Figure 5, which collectively demonstrate that PotF and PlaP are essential for *D. zeae* to uptake exogenous putrescine.

### Disruption of *speA* Alters the Intracellular Putrescine Level

**FIGURE 6:**
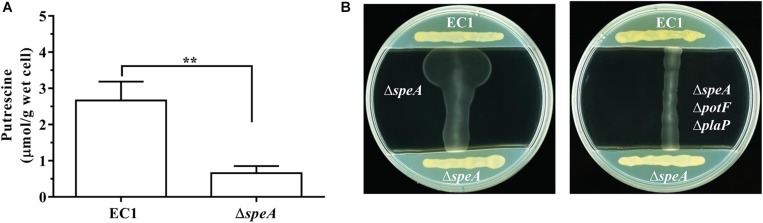
Deletion of *speA* in *D. zeae* strain EC1 decreased cellular level of putrescine and abrogated its ability to promote swimming motility. **(A)** Quantitative measurement of cellular putrescine content in strain EC1 and the *speA* deletion mutant Δ*speA*. The bacterial strains were grown in minimum medium till OD_600_ reached about 1.7. Intracellular putrecine molecules were extracted by freezing and thawing with liquid nitrogen, and benzoylated with benzoyl chloride for analysis with LC-MS. The experiment was repeated three times and error bars denote s.e. **(B)** Putrescine induction of bacterial motility required functional transporters. Strain EC1 and mutant Δ*speA* were grown respectively on the opposite ends of LB plate as indicated to precondition the medium. After 12 h, the middle section of the plate was removed and filled with swimming agar medium. Upon solidification, the mutants Δ*speA* (left panel) and Δ*speA*Δ*potF*Δ*plaP* (right panel) were lined vertically to strain EC1 and the mutant Δ*speA* as indicated. The plates were cultured for 24 h at 28°C before photographing. The experiment was repeated three times with similar results. ^∗∗^*P* < 0.01.

To compare the intracellular concentration of putrescine between wild type EC1 and mutant Δ*speA*, we derivatized the polyamine molecules in bacterial samples by using benzoyl chloride (Lee et al., 2009; Sethi et al., 2011), and determined the concentration of benzoyled putrescine via high-performance liquid chromatography coupled with mass spectrometry (LC-MS). The results showed that the intracellular concentration of putrescine in mutant Δ*speA* was about 4.5-fold lower than that in the wild type EC1 (Figure 6A and Supplementary Figure S5).

To test whether wild type strain could produce and secrete sufficient putrescine signal to induce the swimming motility of the *speA* mutant Δ*speA*, we designed and performed a simple extracellular complementation assay. To a LB plate about 9 cm in diameter, *D. zeae* wild type strain EC1 and the mutant Δ*speA* were streaked on both ends of the plate and incubated for 12 h to precondition the medium with extracellular signals. Then a middle section of the plate about 5 cm in width was removed and replaced with 10 mL of swimming motility medium. After solidify, mutants Δ*speA* and Δ*speA*Δ*potF*Δ*plaP* were streaked respectively on the swimming medium perpendicular to the lines of strain EC1 and mutant Δ*speA* on LB medium, and the plates were cultured at 28°C for 24 h. As showed in Figure 6B, wild type strain EC1 cells were able to induce the swimming motility of mutant Δ*speA*, whereas the mutant Δ*speA* cells were unable to induce itself to swim. Importantly, the extracellular signals from wild type strain EC1 could not restore the swimming motility of the mutant Δ*speA*Δ*potF*Δ*plaP* (Figure 6B), in which the *potF* and *plaP* genes encoding putrescine binding proteins of corresponding putrescine transporters were deleted. The above data suggest that deletion of *speA* abolished the production of signals required for induction of swimming motility in *D. zeae*, and further validate that the putrescine specific transporters are essential for *D. zeae* to respond to extracellular putrescine signals.

### Putrescine Modulates the Transcriptional Expression of Over 200 Genes in *D. zeae* EC1

To determine the scope of putrescine signal influence, we examined the global gene expression profiles of wild type strain EC1 and the *speA* deletion mutant in swimming motility medium with putrescine at the final concentration of 0.1 mM or without using transcriptome analysis. Of the 4,154 genes predicted in EC1 genome (Zhou et al., 2015), expression of 230 genes were significantly (| Fold Change| ≥ 2-fold, *q* ≤ 0.005) influenced by putrescine, including 102 genes upregulated and 128 genes downregulated, which were mostly restored by exogenous addition of putrescine (Supplementary Table S3). These putrescine-dependent genes can be grouped into several functional categories, including (i) secretion systems, (ii) transporter systems, (iii) methyl-accepting chemotaxis proteins, (iv) ribosomal proteins, (v) acyltransferase, and (vi) hypothetical proteins (Supplementary Table S3). The reliability of the transcriptome results was verified by semiquantitative RT-PCR analysis of randomly selected genes listed in Supplementary Table S2 and Supplementary Figure S6. Consistent with the findings that putrescine regulated the bacterial motility and biofilm formation, several genes encoding chemotaxis and flagellar proteins, which are associated with bacterial motility, were downregulated in the *speA* deletion mutant but restored by exogenous addition of putrescine (Supplementary Table S3). In addition, over 10 genes associated with T3SS secretion system were also positively regulated by putrescine (Supplementary Table S3).

### Putrescine Signal Produced by Host Plant Induces the Swimming Motility of *D. zeae*

**FIGURE 7:**
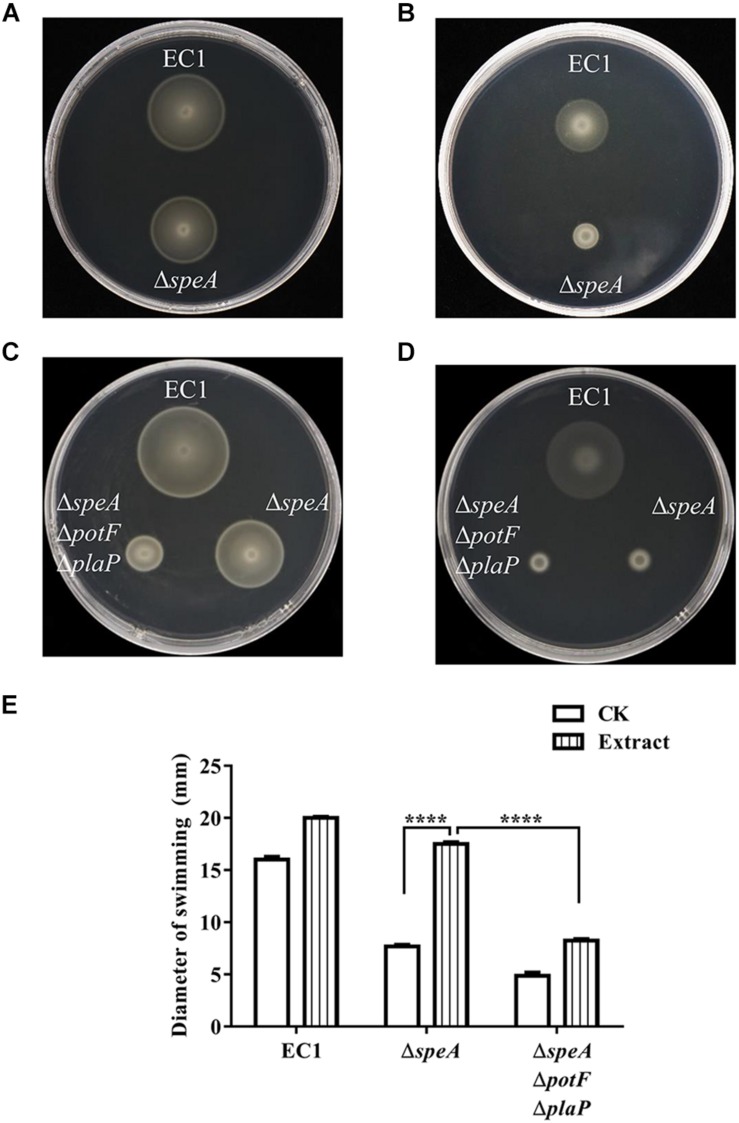
Effect of host cell extract on bacterial swimming motility. The swimming motility of the *D. zeae* strain EC1 and the *speA*-disrupted mutant Δ*speA* in the presence of rice extract **(A)**, and water **(B)**. The swimming motility of strain EC1, mutants Δ*speA* and Δ*speA*Δ*potF*Δ*plaP* in the presence of rice extract **(C)** and water **(D)**. **(E)** Quantitative determination of bacterial cell swimming motility of strain EC1 and its derivatives in the presence of rice extract and water (CK). The data shown are the means ± SE (*n* = 3). ^****^P < 0.0001.

Consistent with the previous report that vigorous growth tissue of plants containing abundant putrescine and other polyamines (Walden et al., 1997), LC-MS analysis indicated that putrescine was present in the rice seedling extract and the total free putrescine concentration was about 27 μM. We then mixed the extract of rice seedling with semi-preparative medium and measured swimming motility of wild type EC1 and its derivatives. After cultured for 12 h with rice seedling extract equivalent to 20 mg of seedling tissue per mL of mixed medium, the swimming motility of the mutant Δ*speA* was significantly increased by about 2.4-fold in comparison with the blank control in which medium was mixed with water (Figures 7A,B,E). In contrast, the swimming motility of strain EC1 and mutant Δ*speA*Δ*potF*Δ*plaP* were also increased by host tissue extract but in a much less extent than the *speA* mutant Δ*speA* (Figures 7C,D,E). The findings indicate that putrescine molecules from host cells could be transported into bacterial cells via putrescine specific transporters and induce the bacterial swimming motility.

### Mutation of *speA* and Transporter Genes in *D. zeae* EC1 Decreases Bacterial Virulence and Invasion Ability on Rice Seeds

**FIGURE 8:**
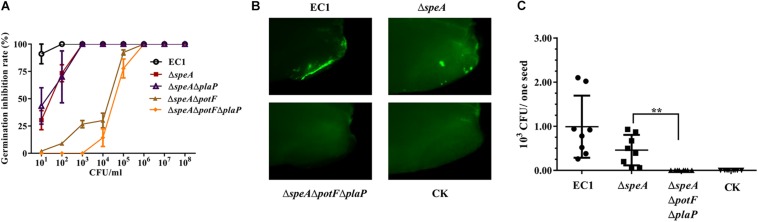
Deletion of *speA* and putrescine transporters attenuated bacterial invasion and virulence of *D. zeae* EC1 on rice seeds. **(A)** Rice seed germination assay. Rice seeds were socked in bacterial suspension containing various amounts of bacterial cells as indicated for 5 h before transferred to plates with moisturized filter papers. The germination rates were counted 5 days later. **(B,C)** Rice seed invasion assay. Strain EC1 and derivatives were labeled with GFP by *in trans* expression of the *gfp* gene. Rice seeds were treated with 100 bacterial cells per mL for 5 h, and then incubated for 24 h at 28°C. The husks of rice seeds were removed for examination under a fluorescence microscope **(B)** and for bacterial CFU counting **(C)**. The representative photographs were presented and the data shown are the means ± SE (*n* = 3). ^∗∗^*P* < 0.01.

To assess the role of putrescine in modulation of bacterial virulence, rice seeds were incubated with various dilutions of *D. zeae* strain EC1 and its derivatives, and the rice seed germination rate were measured 5 days after treatment. The results showed that strain EC1 was highly virulent on rice seeds, showing about 88% inhibition rate when rice seeds were treated with 10 bacterial cells per mL and total inhibition at 100 bacterial cells per mL (Figure 8A). Deletion of *speA* reduced the bacterial virulence by about 35% at 10 bacterial cells per mL and about 13% at 100 bacterial cells (Figure 8A). In contrast, the mutant Δ*speA*Δ*potF*Δ*plaP*, in which both putrescine synthase gene *speA* and transporter genes *potF* and *plaP* were deleted, was unable to inhibit seed germination at a concentration range from 10 to 1000 bacterial cells per mL, and showed merely about 13% inhibition rate when the bacterial inoculum was increased to 10,000 cells per mL (Figure 8A). To test which transporter plays a major role in putrescine-mediated pathogen-host communication, the double deletion mutants Δ*speA*Δ*potF* and Δ*speA*Δ*plaP* were also included in the germination assay. The results clearly indicated that PotF played a dominate role over PlaP in uptaking polyamine signals from host (Figure 8A).

To test how putrescine could influence the bacterial virulence, invasion assay was performed by incubation of rice seeds with the bacterial strains expressing the *gfp* gene at 100 cells per mL. The inoculated seeds with husk removed were examined by fluorescence microscope, and the bacterial cells retained in rice seeds were isolated and measured at 24 h after treatment. The results showed that rice seeds treated with strain EC1 displayed higher intensity of fluorescence than those treated with the mutant Δ*speA*, whereas no fluorescence spot was observed in the seeds treated with the triple-deletion mutant Δ*speA*Δ*potF*Δ*plaP* (Figure 8B). Consistent with the fluorescence analysis, counting bacterial cell numbers invaded into rice seeds showed that on average each seed contained about 1000 strain EC1 cells, less than 500 the mutant Δ*speA* cells, and none the triple mutant *ΔspeAΔpotFΔplaP* cells (Figure 8C).

## Discussion

Bacterial motility and biofilm are important virulence determinants and survival strategies for microbes to cope with harsh environmental conditions and infecting host organisms. In this study, we showed that the deletion of arginine decarboxylase gene *speA* in the plant bacterial pathogen *D. zeae* significantly handicapped its swimming and swarming motility, and drastically attenuated the bacterial biofilm formation ability (Figure 1). The defective phenotypes of the *speA* mutant could be rescued via *in trans* expression of *speA* in mutant or exogenous addition of putresciene or by wild type *D. zeae* EC1 grown at vicinity (Figures 1–4, 6B). This is agreeable with the finding that disruption of *speA* resulted in a drastic reduction of intracellular putrescine level (Figure 6A). Furthermore, we presented evidence that there was no correlation between the defective phenotypes and growth rate, as the growth rate of *speA* mutant was similar to wild type in the defined minimal medium with no polyamines (Supplementary Figure S2). These results demonstrated that SpeA is essential for putrescine biosynthesis and putrescine is required for bacterial cell motility and biofilm formation in *D. zeae*. A similar role of putrescine in regulation of bacterial motility or biofilm formation has also been reported in human bacterial pathogens *P. mirabilis* (Sturgill and Rather, 2004), *Y. pesti* (Patel et al., 2006; Wortham et al., 2010), and environmental isolate *S. oneidensis* (Ding et al., 2014). The identified roles of putrescine in the plant pathogen *D. zeae* thus further validate its conserved role in modulation of bacterial motility and biofilm formation in diverse microorganisms. The data of transcriptome analysis suggest that putrescine might modulate bacterial motility through regulation of bacterial chemotaxis and flagellar biogenesis (Supplementary Table S3), as bacterial movement is guided and driven by chemotaxis and flagellar, respectively (Guttenplan and Kearns, 2013; Jones and Armitage, 2015).

Bacteria require either a polyamine influx transporter or a sensor when extracellular polyamines are used as growth factors or signaling molecules (Furuchi et al., 1991; Karatan et al., 2005; Zhou et al., 2007). This is because polyamines are hydrophilic molecules that cannot passively diffuse across that bacterial plasma membrane. In this work, we found two putrescine specific transporters in *D. zeae* strain EC1, represented by their cognate putrescine binding proteins PlaP and PotF, respectively, which collectively play key roles for influx transportation of exogenous putrescine into bacterial cells (Figures 5, 8 and Supplementary Figure S3). Significantly, our results showed unequivocally that two transporters, in particular the one represented by PotF, are essential for influxing extracellular putrescine signals either from *D. zeae* itself or from host plant (Figures 6–8). Identification of two putrescine influx transporters further validates the important role of putrescine as a signal for bacterial cell-cell communication and for pathogen-host interactions.

Interestingly, two transporters appeared to act differently in modulation of bacterial motility when assayed in the presence or absence of exogenous putrescine. In the absence of exogenous putrescine, deletion of *potF* in the genetic background of *speA* mutant resulted in reduced bacterial swimming motility to a level similar to the *speA/potF/plaP* triple-deletion mutant Δ*speA*Δ*potF*Δ*plaP*, whereas further deletion of *plaP* in the mutant Δ*speA* caused only a minor reduction in motility compared to its parental strain Δ*speA* (Supplementary Figure S3). This suggests that PotF transporter plays a dominant role in regulation of bacterial biological motility. In contrast, however, in the presence of exogenous putrescine, complementation of the *speA/potF/plaP* triple-deletion mutant Δ*speA*Δ*potF*Δ*plaP* with *plaP* could fully restore the bacterial motility in a capacity even superior than *potF* (Figure 5). Similarly, motility assay of double-deletion mutants Δ*speA*Δ*potF* and Δ*speA*Δ*plaP* further confirmed the superior performance of PlaP transporter over PotF with exogenous addition of putrescine (Supplementary Figure S3E). The contrasting performance of PotF and PlaP transporters with or without exogenous putrescine may suggest different sensitivities of two transporters to putrescine, either at transcriptional or translational level or both, which awaits further investigations.

In addition to its role as a bacterial intercellular signal, the results from this study also indicate that putrescine plays a key role in pathogen-host interactions. Putrescine is present in both bacterial and plant cells (Igarashi and Kashiwagi, 2000), but it is not clear whether its level in plant cells is sufficiently high enough to influence the physiological processes of bacterial cells. By using a combination of putrescine derivatization and HPLC-MS analysis, we showed that rice seeds in germination contained about 27 μM putrescine. Given that exogenous addition of 10 μM putrescine could substantially induce bacterial swimming motility (Figure 3A), we argued that *D. zeae* could exploit the putrescine molecules produced by rice host as a cross-kingdom communication signal to promote bacterial motility and systemic infection. The notion is greatly strengthened by the observation that deletion of the putrescine binding proteins of cognate transporters significantly reduced the host cell extract-induced swimming motility (Figure 7C). The role of putrescine in host-pathogen interaction is reminiscent to the findings of spermidine in the human bacterial pathogen *P. aeruginosa*. It was shown previously that the spermidine produced by mammalian host could be exploited by *P. aeruginosa* to trigger the transcriptional expression of type III secretion system (T3SS) genes via a polyamine transporter (Zhou et al., 2007), although it remains to be determined whether *P. aeruginosa* could also produce sufficient amount of spermidine molecules and utilize these molecules as an intercellular signal to influence T3SS expression. Similarly, transcriptome analysis indicated that putrescine might also play a role in regulation of T3SS expression in *D. zeae* EC1 (Supplementary Table S3), which awaits further investigations. The findings from this study add one more polyamine molecule as an effective and potent cross-kingdom communication signal in modulation of bacterial physiological activity. It remains to be investigated whether host could induce putrescine biosynthesis in *D. zeae* or the bacterial infection might trigger host plant to produce higher level of putrescine than the untreated rice seeds. A recent study showed that putrescine was enriched 76-fold to 37 μM in *R. solanacearum*-infected sap of tomato xylem vessels, and treatment of plants with 500 μM putrescine before inoculation accelerated wilt symptom development and bacterial growth and spread (Lowe-Power et al., 2018). Consistent with the latter option, the same study also showed that *R. solanacearum* infection induced expression of several plant putrescine biosynthesis genes (Lowe-Power et al., 2018).

Cell motility is closely associated with bacterial pathogenesis and virulence, which has been documented in *Ralstonia solanacearum* (Tans-Kersten et al., 2001), and *Proteus mirabilis* (Allison et al., 1994; Kurihara et al., 2013). Given this important function, it seems highly logic and rational that *D. zeae* has involved mechanisms allowing utilization of putrescine signals from bacterium itself and host plant in modulation of virulence. A clear cumulative effect of putrescine biosynthesis and uptaking on bacterial motility and virulence was shown when *speA* single deletion mutant and *speA/potF* douple mutant and *speA/potF/plaP* triple mutant were compared (Supplementary Figure S3E and Figure 8A). Interestingly, deletion of *speA* and its transporter genes *potF* and *plaP* did not affect biosynthesis of zeamines (data not shown), which are known phytotoxins and inhibitors on rice seed germination (Zhou et al., 2011; Cheng et al., 2013). To explore the mechanism that links motility and virulence, we used *D. zeae* EC1 and its derivatives expressing *gfp* to infect rice seeds, and the results showed that the pathogen failed to penetrate and invade rice seeds when putrescine production and transportation were blocked (Figures 8B,C). This suggests that for optimum toxic effect, phytotoxin zeamines should be produced inside rice seeds. Intriguingly, *D. zeae* produces only a low level of zeamines about 7 μM per mL in minimum medium (Liao et al., 2014), but total inhibition of rice seed germination requires a high concentration of purified zeamines (> 480 μM) (Zhou et al., 2011). This is apparently inconsistent with the finding that inoculation with only a few bacterial cells (about 10 cells per mL) could abrogate rice seed germination (Figure 8A; Hussain et al., 2008). The results from this study explain this intriguing puzzle and provide useful clues and new targets for designing and developing new approaches to prevent and control bacterial infections.

## Data Availability

This manuscript contains previously unpublished data. The raw data of transcriptome analyses were available under SRA accession number PRJNA516290.

## Author Contributions

L-HZ and ZS conceived and designed the experiments. ZS, QW, YL, and ZL performed the experiments. LX, JZ, and ZC analyzed the data. ZS and L-HZ wrote the manuscript. All authors read and approved the final manuscript.

## Conflict of Interest Statement

The authors declare that the research was conducted in the absence of any commercial or financial relationships that could be construed as a potential conflict of interest.
