# Supplementary material for: Putrescine Is an Intraspecies and Interkingdom Cell-Cell Communication Signal Modulating the Virulence of Dickeya zeae
Source: Front Microbiol. 2019 Aug 21;10:1950. doi: 10.3389/fmicb.2019.01950 (PMC6712546; doi:10.3389/fmicb.2019.01950)
Supplement: Supplementary file 1 [file Data_Sheet_1.docx]

**Supplementary Material**

Shi et al., Putrescine is an intraspecies and interkingdom cell-cell communication signal modulating the virulence of *Dickeya zeae*

**Content:**

**Supplementary Table S1**. Bacterial strains and plasmids used in this study.

**Supplementary Table S2.** Primers used in this study.

**Supplementary Table S3.** List of the genes regulated by putrescine signal.

**Supplementary Figure S1.** Effects of disruption enzymes of polyamine biosynthesis on cell motility and biofilm formation.

**Supplementary Figure S2.** Growth curves of *Dickeya zeae* EC1 and its derivatives in the minimal medium.

**Supplementary Figure S3.** Influence of putrescine transporters on swimming motility.

**Supplementary Figure S4.** Effect of exogenous addition of polyamines on bacterial swimming motility.

**Supplementary Figure S5.** High-performance liquid chromatography coupled with mass spectrometry (LC-MS) detection the concentration of benzoyled putrescine of *Dickeya zeae* EC1 and the *speA*-disrupted mutant ∆*speA* cells.

**Supplementary Figure S6.** RT-PCR analysis of putrescine signal on modulating genes expression.

**Supplementary Table S1.** Strains and plasmids used in this study

| **Strains or plasmid** | **Relevant phenotypes and characteristics^a^** | **Source or reference** |
| --- | --- | --- |
| ***Dickeya zeae*** |  |  |
| EC1 | Wild type of *Dickeya zeae*, Pmb ^r^ | Lab collection |
| ∆*speA* | *speA* deletion mutant derived from EC1 | This study |
| ∆*speA*∆potF∆*plaP*  ∆*speC*  ∆*speE*  ∆*speA*∆*potF*  ∆*speA*∆*plaP*  ∆*speA*(*speA*)  ∆*speA*∆*potF*∆*plaP*(*potF*)  ∆*speA*∆*potF*∆*plaP*(*plaP*)  ∆*speA*∆*potF*(*potF*) | *potF* and *plaP* deletion mutant derived from ∆*speA*  *speC* deletion mutant derived from EC1  *speE* deletion mutant derived from EC1  *potF* deletion mutant derived from ∆*speA*  *plaP* deletion mutant derived from ∆*speA*  The complemented strain of ∆*speA*, Gm^r^  The complemented strain of ∆*speA*∆*potF*∆*plaP*, Amp^r^  The complemented strain of ∆*speA*∆*potF*∆*plaP*, Amp^r^  The complemented strain of ∆*speA∆potF*, Amp^r^ | This study  This study  This study  This study  This study  This study  This study  This study  This study |
| ***Escherichia coli*** |  |  |
| CC118 | Host for plasmid constructs derived from pKNG101 | Lab collection |
| DH5α | Host for plasmid constructs derived from pBBRI-MCS4 | Lab collection |
| pRK2013 | *Thr leu thi recA hsdR hsdM pro,* Km^r^ | Lab collection |
| **Plasmids** |  |  |
| pKNG101 | Knockout vector, Str^r^ | Lab collection |
| pKNG101-*speA*  pKNG101-*speC*  pKNG101-*speE*  pKNG101-*potF*  pKNG101- *plaP* | pKNG101 containing in-frame deleted fragement of *speA*, Str^r^  pKNG101 containing in-frame deleted fragement of *speC*, Str^r^  pKNG101 containing in-frame deleted fragement of *speE*, Str^r^  pKNG101 containing in-frame deleted fragement of *potF*, Str^r^  pKNG101 containing in-frame deleted fragement of *plaP*, Str^r^ | This study  This study  This study  This study  This study |
| pBBRI-MCS4 | Multi-copy expression vector, Gm^r^ | Lab collection |
| pBBRI- *speA*  pBBRI- *potF*  pBBRI- *plaP* | pBBRI-MCS4 containing *speA* encoding region at the downstream of *lac* promoter, Gm^r^  pBBRI-MCS4 containing *potF* encoding region at the downstream of *lac* promoter, Amp^r^  pBBRI-MCS4 containing *plaP* encoding region at the downstream of *lac* promoter, Amp^r^ | This study  This study  This study |

^a^ Pmb ^r^, Gm^r^, Amp^r^, Km^r^, or Str^r^ = resistance to polymyxin B, gentamycin, ampicillin, kanamycin, or streptomycin, respectively.

**Supplementary Table S2.** Primers used in this study

| Primer | Decription^a^ | Sequence | |
| --- | --- | --- | --- |
| A-1 | Forward primer for upstream of *speA* | 5’-CTGATGGTTGTCGTGATAGC-3’ |  |
| A-2 | Reverse primer for upstream of *speA* | 5’-CTCATCAAGGAACTGTGCCTCATTCATGGCTACCTCCTG-3’ |  |
| A-3 | Forward primer for downstream of *speA* | 5’-CAGGAGGTAGCCATGAATGAGGCACAGTTCCTTGATGAG-3’ |  |
| A-4  C-1  C-2  C-3  C-4  E-1  E-2  E-3  E-4 | Reverse primer for downstream of *speA*  Forward primer for upstream of *speC*  Reverse primer for upstream of *speC*  Forward primer for downstream of *speC*  Reverse primer for downstream of *speC*  Forward primer for upstream of *speE*  Reverse primer for upstream of *speE*  Forward primer for downstream of *speE*  Reverse primer for downstream of *speE* | 5’-GAAGTACTGAGCGATATCAC-3’  5’-TGATGTCTATCGGACGGTG-3’  5’-ACGCTGTTCATGCTTGCGATACAAACGAGCCTGATACTCC-3’  5’-GGAGTATCAGGCTCGTTTGTATCGCAAGCATGAACAGCGT-3’  5’-GTCTGATGACCAATTAGCGA-3’  5’-TTGAACAAACAGCACCTCG-3’  5’-GCTCTGTTGGTCGATAGCAGACATGTTAGGGCTTCCT-3’  5’-AGGAAGCCCTAACATGTCTGCTATCGACCAACAGAGC-3’  5’-ATAGGTGTGCACACAGATGT-3’ |  |
| potF-1 | Forward primer for upstream of *potF* | 5’-TGCTGAACGATTCAACACCT-3’ |  |
| potF -2 | Reverse primer for upstream of *potF* | 5’-GGTCCACGAGCGTGTAATCAACCAGTTATAGACGTGCAGC-3’ |  |
| potF -3 | Forward primer for downstream of *potF* | 5’-GCTGCACGTCTATAACTGGTTGATTACACGCTCGTGGACC-3’ |  |
| potF -4 | Reverse primer for downstream of *potF* | 5’-ACCAGCGTTAGCATCTCTTC-3’ |  |
| plaP-1 | Forward primer for upstream of *plaP* | 5’-GAACGTGGATTACAGCAGGT-3’ |  |
| plaP-2 | Reverse primer for upstream of *plaP* | 5’-TCTTCGTTGTACTGCGGTACTGTGGAAACCGTGGAATCAG-3’ |  |
| plaP-3 | Forward primer for downstream of *plaP* | 5’- CTGATTCCACGGTTTCCACAGTACCGCAGTACAACGAAGA-3’ |  |
| plaP-4 | Reverse primer for downstream of *plaP* | 5’-TAGCGTAAGAGAGAAAGTCG-3’ |  |
| HB-A-F | Forward primer for encoding region of *speA* | 5’-CTGCGTCACTGACTCAATC-3’ |  |
| HB-A-R | Reverse primer for encoding region of *speA* | 5’- ACCGTACAGCAGTCAGTATG-3’ |  |
| HB-potF-F | Forward primer for encoding region of *potF* | 5’-GATCAATAGCTGGTCATCAG-3’ |  |
| HB-potF-R | Reverse primer for encoding region of *potF* | 5’-GCATGGAAGTGGAGTCAAGG-3’ |  |
| HB-plaP-F | Forward primer for encoding region of *plaP* | 5’-CGCTGTTGATGGTTCCGGAT-3’ |  |
| HB-plaP-R | Reverse primer for encoding region of *plaP* | 5’-CCTATGCACAACAACTGCTG-3’ |  |
| 16F | Forward primer for RT-PCR of 16S rRNA | 5’-GGCCTAACACATGCAAGTCG-3’ |  |
| 16R | Reverse primer for RT-PCR of 16S rRNA | 5’-GAGTTAGCCGGTGCTTCTTC-3’ |  |
| 1 F | Forward primer for RT-PCR of gene 1 | 5’-CCCAGGCAAAAGTCATGATCCC-3’ |  |
| 1 R | Reverse primer for RT-PCR of gene 1 | 5’-ACGAAATGATGACCCACGTTC-3’ |  |
| 2 F | Forward primer for RT-PCR of gene 2 | 5’-GGCTTTCCGGCAATCAATCCAGT-3’ |  |
| 2 R | Reverse primer for RT-PCR of gene 2 | 5’-GAGATCCTCCAGACCGCCTGT-3’ |  |
| 3 F | Forward primer for RT-PCR of gene 3 | 5’-TTCGCCCAGGATTTGTCGTCT-3’ |  |
| 3 R | Reverse primer for RT-PCR of gene 3 | 5’-ACCCGTTGTCCGACATGTTCC-3’ |  |
| 4 F | Forward primer for RT-PCR of gene 4 | 5’-GTACATTCGCCACCCGCTCA-3’ |  |
| 4 R | Reverse primer for RT-PCR of gene 4 | 5’-CAGGCCATTGAACGTAATGCT-3’ |  |
| 5 F | Forward primer for RT-PCR of gene 5 | 5’-AGTCCATCTGCATATCCGTGA-3’ |  |
| 5 R | Reverse primer for RT-PCR of gene 5 | 5’-GCGATGACCAGTATGAAATACCT-3’ |  |
| 6 F | Forward primer for RT-PCR of gene 6 | 5’-CTTCGCCGTGATCAATCCAGA-3’ |  |
| 6 R | Reverse primer for RT-PCR of gene 6 | 5’-CCAAGTTCACGCTGGAAATCGC-3’ |  |
| Primer | Decription^a^ | Sequence |  |
| 7F | Forward primer for RT-PCR of gene 7 | 5’-TGGTATTCAGTACTTCCACGTT-3’ |  |
| 7R | Reverse primer for RT-PCR of gene 7 | 5’-CTTATCGCGCCAAACAGGT-3’ |  |
| 8F | Forward primer for RT-PCR of gene 8 | 5’-CTGTTTGTCGTACGCCGTTC-3’ |  |
| 8R | Reverse primer for RT-PCR of gene 8 | 5’-CAAGTTGCCACGCTCGAAA-3’ |  |
| 9F | Forward primer for RT-PCR of gene 9 | 5’-CCAGTCCGCTGTGATCAACGA-3’ |  |
| 9R | Reverse primer for RT-PCR of gene 9 | 5’-TGTTGATGCTGTCTACGCTCA-3’ |  |
| 10F | Forward primer for RT-PCR of gene 10 | 5’-CCTGCCGGTATTCATGGTCA-3’ |  |
| 10R | Reverse primer for RT-PCR of gene 10 | 5’-AGAACCACGTTGTCTTCGAGT-3’ |  |
| 11F | Forward primer for RT-PCR of gene 11 | 5’-ACCGGCTTCGATACCAATTCCC-3’ |  |
| 11R | Reverse primer for RT-PCR of gene 11 | 5’-GCCAGCATCACCGAGCCAT-3’ |  |
| 12F | Forward primer for RT-PCR of gene 12 | 5’-CGTACTCGCTGTATTCGCACT-3’ |  |
| 12R | Reverse primer for RT-PCR of gene 12 | 5’-CCTGCGTGATCTCCTCGTT-3’ |  |
| 13F | Forward primer for RT-PCR of gene 13 | 5’-CAGCGTGACAGCAAACTCC-3’ |  |
| 13R | Reverse primer for RT-PCR of gene 13 | 5’-CAAGAAATTCGCGGTTACGAT-3’ |  |

^a^ Gene ID of gene 1, 2, 3, 4, 5, 6, 7, 8, 9, 10, 11, 12 and 13 is W909_RS04875, W909_RS09730, W909_RS10380, W909_RS10395, W909_RS10435, W909_RS10550, W909_RS12085, W909_RS12490, W909_RS13025, W909_RS13030, W909_RS18920, W909_RS17140 and W909_RS12150, respectively.

**Supplementary Table S3.** List of the genes regulated by putrescine^a^

| Gene ID | Fold change  (∆*speA*/∆*speA*+Put) | Pval | Fold change  (EC1/∆*speA*) | Pval | Function |
| --- | --- | --- | --- | --- | --- |
| **Chemotaxis and flagellar protein** | |  |  |  |  |
| W909_RS18925 | -2.1 | 1.06E-68 | 2.9 | 4.00E-156 | Methyl-accepting chemotaxis protein |
| W909_RS11860 | -1.7 | 1.05E-65 | 2.3 | 1.08E-132 | Methyl-accepting chemotaxis protein |
| W909_RS12085 | 2.2 | 4.11E-23 | -1.2 | 9.14E-10 | flgC flagellar basal-body rod protein FlgC |
| W909_RS09590 | 1.8 | 4.93E-80 | -1.5 | 1.17E-60 | Methyl-accepting chemotaxis protein |
| W909_RS18920 | 2.2 | 1.69E-115 | -1.7 | 1.05E-80 | Methyl-accepting chemotaxis protein |
| W909_RS17140 | 1.9 | 3.47E-47 | -1.7 | 9.03E-40 | Methyl-accepting chemotaxis protein |
| W909_RS12150 | 1.8 | 1.77E-85 | -1.6 | 2.39E-70 | chemotaxis protein CheW |
| **Ribosomal protein** | |  |  |  |  |
| W909_RS15750 | -3 | 0 | 3.5 | 0 | 30S ribosomal protein |
| W909_RS16720 | -2.2 | 3.56E-166 | 2.5 | 3.44E-223 | 30S ribosomal protein |
| W909_RS15740 | -3.1 | 4.09E-28 | 3.4 | 1.42E-35 | 30S ribosomal protein |
| W909_RS03025 | -2.3 | 4.61E-235 | 2.6 | 4.96E-306 | 30S ribosomal protein |
| W909_RS04745 | -2.7 | 0 | 2.8 | 0 | 30S ribosomal protein |
| W909_RS17575 | -1.9 | 3.30E-117 | 2.1 | 4.95E-129 | 30S ribosomal protein |
| W909_RS17620 | -2.2 | 3.72E-52 | 2.2 | 2.27E-45 | 30S ribosomal protein |
| W909_RS17645 | -2.7 | 0 | 2.9 | 0 | 30S ribosomal protein |
| W909_RS17670 | -2.4 | 0 | 2.5 | 0 | 30S ribosomal protein |
| W909_RS17675 | -2.4 | 0 | 2.6 | 0 | 30S ribosomal protein |
| W909_RS20460 | -2.3 | 0 | 2.4 | 0 | 50S ribosomal protein |
| W909_RS17550 | -2.8 | 0.00036983 | 2.9 | 0.00021233 | 50S ribosomal protein |
| W909_RS17580 | -1.8 | 9.69E-230 | 2 | 1.03E-289 | 50S ribosomal protein |
| W909_RS17585 | -1.9 | 7.10E-90 | 2.1 | 8.09E-111 | 50S ribosomal protein |
| W909_RS17590 | -1.9 | 0 | 2.2 | 0 | 50S ribosomal protein |
| W909_RS17600 | -2.7 | 2.34E-07 | 2.4 | 2.31E-05 | 50S ribosomal protein |
| W909_RS17615 | -2.3 | 3.16E-89 | 2 | 9.47E-63 | 50S ribosomal protein |
| W909_RS17625 | -2.1 | 0 | 2 | 0 | 50S ribosomal protein |
| W909_RS17630 | -2.4 | 2.30E-75 | 2.5 | 1.27E-73 | 50S ribosomal protein |
| W909_RS17635 | -2.3 | 3.87E-280 | 2.5 | 6.06E-305 | 50S ribosomal protein |
| W909_RS17640 | -2.6 | 0 | 2.8 | 0 | 50S ribosomal protein |
| W909_RS18665 | -2.2 | 0 | 2.4 | 0 | 50S ribosomal protein |
| W909_RS00705 | -3 | 0 | 3.2 | 0 | 50S ribosomal protein |
| W909_RS00710 | -3.3 | 2.97E-10 | 3.8 | 1.53E-14 | 50S ribosomal protein |
| W909_RS01065 | -2.4 | 0 | 2.6 | 0 | 50S ribosomal protein |
| W909_RS01070 | -2.3 | 0 | 2.5 | 0 | 50S ribosomal protein |
| W909_RS01075 | -2.5 | 0 | 2.5 | 0 | 50S ribosomal protein |
| W909_RS01080 | -2.2 | 0 | 2.1 | 1.93E-260 | 50S ribosomal protein |
| W909_RS01545 | -2.2 | 0 | 2.2 | 0 | 50S ribosomal protein |
| W909_RS02930 | -1.8 | 1.41E-263 | 2 | 0 | 50S ribosomal protein |
| W909_RS04760 | -2.7 | 0 | 2.6 | 0 | 50S ribosomal protein |
| Gene ID | Fold change  (∆*speA*/∆*speA*+Put) | Pval | Fold change  (EC1/∆*speA*) | Pval | Function |
| W909_RS07890 | -3.6 | 0 | 3.9 | 0 | 50S ribosomal protein |
| W909_RS11735 | -3.2 | 3.13E-93 | 3.4 | 1.49E-112 | 50S ribosomal protein L3 |
| W909_RS15735 | -2.6 | 3.24E-167 | 2.9 | 5.80E-210 | 50S ribosomal protein |
| **NADH dehydrogenase** | |  |  |  |  |
| W909_RS12920 | 3.2 | 6.90E-88 | -2.5 | 2.31E-67 | NADH-quinone oxidoreductase subunit N |
| W909_RS12925 | 3 | 4.40E-90 | -2.2 | 1.27E-64 | NADH-quinone oxidoreductase subunit M |
| W909_RS12930 | 2.9 | 7.09E-94 | -2.2 | 9.38E-66 | NADH-quinone oxidoreductase subunit L |
| W909_RS12935 | 2.8 | 0.00032106 | -3.1 | 0.00011755 | NADH-quinone oxidoreductase subunit K |
| W909_RS12940 | 3 | 3.17E-24 | -3.3 | 5.54E-27 | NADH-quinone oxidoreductase subunit J |
| W909_RS12950 | 2.8 | 1.11E-38 | -2 | 2.02E-26 | NADH-quinone oxidoreductase subunit H |
| **Transporters** | |  |  |  |  |
| W909_RS16030 | -2.2 | 1.51E-16 | 3.2 | 8.71E-43 | Aromatic amino acid transporter |
| W909_RS01245 | -2.7 | 5.86E-22 | 3.8 | 6.42E-54 | Amino acid ABC transporter substrate-binding protein |
| W909_RS04140 | 2.5 | 1.02E-20 | -2 | 3.63E-16 | Iron ABC transporter substrate-binding protein |
| W909_RS06460 | 1.7 | 0.0023218 | -2.7 | 5.97E-06 | Iron permease |
| W909_RS06465 | 2.5 | 5.54E-21 | -3 | 8.83E-27 | Iron uptake system protein EfeO |
| W909_RS07710 | 3 | 7.83E-44 | -2.5 | 2.54E-37 | Iron ABC transporter substrate-binding protein |
| W909_RS07715 | 2 | 5.27E-13 | -2.4 | 1.30E-16 | Iron ABC transporter permease |
| W909_RS07840 | -2 | 8.60E-13 | 2.4 | 3.10E-18 | ATP-binding protein |
| W909_RS10550 | -1.9 | 1.10E-17 | 2.8 | 1.36E-41 | ABC transporter substrate-binding protein |
| W909_RS14360 | 3.2 | 1.71E-56 | -2.2 | 1.70E-37 | ABC transporter substrate-binding protein |
| W909_RS14365 | 2.5 | 1.86E-24 | -2.3 | 5.54E-23 | Iron ABC transporter permease |
| W909_RS14370 | 2.5 | 1.17E-22 | -2.5 | 9.52E-23 | ABC transporter ATP-binding protein |
| W909_RS19660 | 1.3 | 2.32E-05 | -2.4 | 4.97E-14 | Amino acid ABC transporter permease |
| W909_RS06565 | -2 | 1.84E-23 | 2.1 | 3.90E-23 | Efflux RND transporter periplasmic adaptor subunit |
| W909_RS06785 | 2.2 | 9.12E-13 | -3.1 | 1.51E-19 | Cyclic peptide export ABC transporter |
| W909_RS08320 | -2.3 | 2.53E-68 | 2.2 | 2.43E-61 | MFS transporter |
| **t-RNA** | |  |  |  |  |
| W909_RS01040 | -2.2 | 0.0014186 | 2.7 | 3.95E-05 | tRNA-Gly |
| W909_RS02990 | -4 | 0.0026663 | 3.8 | 0.0080162 | tRNA-Leu |
| W909_RS02995 | -5 | 0.0068343 | 5.6 | 0.001274 | tRNA-Met |
| W909_RS03150 | -4.6 | 0.00081017 | 5.1 | 0.00012696 | tRNA-Leu |
| W909_RS03155 | -4.5 | 2.15E-11 | 4.5 | 9.27E-11 | tRNA-Leu |
| W909_RS03260 | -1.9 | 3.48E-09 | 2 | 5.81E-09 | tRNA-Gly |
| W909_RS04340 | -4.7 | 0.00071586 | 4.8 | 0.00063242 | tRNA-Arg |
| W909_RS04345 | -5.8 | 4.88E-07 | 5.6 | 5.21E-06 | tRNA-Arg |
| W909_RS09060 | -2.7 | 4.32E-11 | 2.3 | 2.40E-07 | tRNA-Tyr |
| W909_RS12480 | -2.7 | 2.07E-18 | 2.6 | 7.53E-16 | tRNA-Lys |
| W909_RS12495 | -2.6 | 2.32E-05 | 2.4 | 0.00029397 | tRNA-Asn |
| W909_RS13475 | -4.2 | 3.96E-14 | 4 | 1.34E-11 | tRNA-Arg |
| W909_RS14580 | -3.3 | 0.00090719 | 2.8 | 0.010168 | tRNA-Ala |
| Gene ID | Fold change  (∆*speA*/∆*speA*+Put) | Pval | Fold change  (EC1/∆*speA*) | Pval | Function |
| W909_RS17065 | -4.1 | 6.26E-33 | 4.6 | 2.75E-44 | tRNA-Gly |
| W909_RS17075 | -3.2 | 3.86E-15 | 3 | 1.99E-12 | tRNA-Gly |
| W909_RS17300 | -2.6 | 0.0030994 | 2.2 | 0.019273 | tRNA-Gly |
| **Transcriptional regulator** | |  |  |  |  |
| W909_RS00370 | -3.8 | 1.36E-208 | 3.9 | 7.61E-215 | AraC family transcriptional regulator |
| W909_RS05715 | -2.4 | 7.36E-16 | 2.4 | 7.10E-16 | LexA regulated protein |
| W909_RS12490 | -3.9 | 3.26E-75 | 4.5 | 4.42E-119 | LysR family transcriptional regulator |
| W909_RS09755 | 2.2 | 4.44E-292 | -3.1 | 0 | Fatty acid metabolism transcriptional regulator FadR |
| W909_RS12555 | 1.9 | 5.81E-09 | -2.1 | 1.48E-10 | Transcriptional regulator |
| **Hypothetical protein** | |  |  |  |  |
| W909_RS13825 | 3.1 | 1.10E-33 | -2.2 | 2.28E-22 | DUF4879 domain-containing protein |
| W909_RS10435 | 5.2 | 0 | -5.5 | 0 | Hypothetical protein |
| W909_RS10400 | 2.3 | 0.0027978 | -3.2 | 0.00024721 | Hypothetical protein |
| W909_RS09730 | 6.4 | 5.29E-165 | -7.1 | 5.84E-160 | Hypothetical protein |
| W909_RS09735 | 2.8 | 5.51E-11 | -3.3 | 5.76E-13 | Hypothetical protein |
| W909_RS10420 | 1.8 | 0.0016504 | -2.3 | 8.58E-05 | Hypothetical protein |
| W909_RS10485 | 5.8 | 9.92E-06 | -3.5 | 0.00010593 | Hypothetical protein |
| W909_RS14095 | 2.6 | 9.31E-14 | -2.6 | 3.20E-14 | DUF1365 domain-containing protein |
| W909_RS14105 | 2.7 | 3.70E-07 | -3 | 5.87E-08 | DUF3833 domain-containing protein |
| W909_RS14110 | 2.7 | 1.89E-10 | -2.8 | 1.85E-11 | DUF1722 domain-containing protein |
| W909_RS14130 | 1.9 | 4.12E-38 | -2.2 | 5.00E-48 | Hypothetical protein |
| W909_RS17200 | 2.8 | 9.87E-82 | -2.4 | 9.71E-69 | Hypothetical protein |
| W909_RS17215 | 4.4 | 1.52E-71 | -4 | 8.17E-68 | Hypothetical protein |
| W909_RS17220 | 4.6 | 9.47E-244 | -4 | 5.77E-223 | Hypothetical protein |
| W909_RS17255 | 3.3 | 1.12E-209 | -2.8 | 1.31E-180 | Hypothetical protein |
| W909_RS20130 | 5.5 | 0 | -4.2 | 0 | DUF4879 domain-containing protein |
| W909_RS06180 | -3.6 | 0 | 2.1 | 8.14E-82 | Hypothetical protein |
| W909_RS06275 | -3.9 | 5.09E-25 | 4 | 1.71E-25 | DUF4123 domain-containing protein |
| W909_RS02510 | -2.3 | 1.85E-11 | 2.2 | 1.47E-09 | Hypothetical protein |
| W909_RS00580 | -2 | 1.21E-05 | 2.2 | 2.37E-06 | Hypothetical protein |
| W909_RS00400 | -2.2 | 1.71E-100 | 2.5 | 2.56E-135 | Hypothetical protein |
| W909_RS06280 | -3.3 | 3.20E-17 | 3.6 | 3.62E-21 | DUF4123 domain-containing protein |
| W909_RS07390 | -2.9 | 1.90E-20 | 2.5 | 2.26E-13 | Hypothetical protein |
| W909_RS08875 | -5.5 | 0 | 3.4 | 1.68E-116 | Hypothetical protein |
| W909_RS12505 | -2.4 | 6.39E-08 | 2.8 | 7.30E-11 | DUF736 domain-containing protein |
| W909_RS12530 | -2.3 | 4.21E-87 | 2.1 | 1.26E-67 | Hypothetical protein |
| W909_RS12535 | -3.4 | 1.81E-39 | 2.9 | 9.87E-26 | DUF4123 domain-containing protein |
| W909_RS15595 | -2.6 | 3.43E-07 | 2.8 | 2.23E-08 | Hypothetical protein |
| W909_RS17050 | -3 | 5.97E-64 | 2.5 | 3.91E-37 | Hypothetical protein |
| W909_RS20105 | -2.4 | 0.0057339 | 2.2 | 0.01687 | Hypothetical protein |
| W909_RS20115 | -4.9 | 3.66E-48 | 3.4 | 5.83E-15 | Hypothetical protein |
| W909_RS20525 | -1.8 | 1.56E-05 | 2.1 | 7.97E-07 | Hypothetical protein |
| Gene ID | Fold change  (∆*speA*/∆*speA*+Put) | Pval | Fold change  (EC1/∆*speA*) | Pval | Function |
| **Type III secretion** | |  |  |  |  |
| W909_RS09720 | 3 | 1.24E-16 | -3.1 | 1.72E-17 | AvrF family protein |
| W909_RS09725 | 3.7 | 1.01E-162 | -4.2 | 1.39E-178 | AvrE family type 3 secretion system effector |
| W909_RS10380 | 5.4 | 2.35E-235 | -6 | 6.97E-238 | Harpin hrpN |
| W909_RS10385 | 2.3 | 0.00092433 | -3.3 | 2.50E-05 | Hypothetical protein |
| W909_RS10395 | 3.3 | 5.99E-38 | -3.5 | 1.56E-40 | EscC family type III secretion system outer membrane ring protein |
| W909_RS10425 | 2.2 | 1.65E-08 | -2.1 | 5.75E-08 | EscJ family type III secretion inner membrane ring protein |
| W909_RS10460 | 3.8 | 2.28E-18 | -3.3 | 1.03E-16 | TyeA family type III secretion system gatekeeper subunit |
| W909_RS10465 | 2.4 | 4.88E-08 | -3.6 | 2.46E-12 | EscV family type III secretion system export apparatus protein |
| W909_RS10470 | 3.4 | 2.42E-08 | -3.8 | 4.08E-09 | EscD family type III secretion system inner membrane ring protein |
| W909_RS10475 | 2.6 | 5.34E-06 | -3.6 | 3.30E-08 | EscN/YscN/HrcN family type III secretion system ATPase |
| W909_RS10490 | 3.5 | 1.65E-15 | -3.7 | 1.26E-16 | YscQ/HrcQ family type III secretion apparatus protein |
| W909_RS10495 | 3 | 0.00039554 | -3.7 | 6.79E-05 | EscR family type III secretion system export apparatus protein |
| **Type VI secretion** | |  |  |  |  |
| W909_RS06250 | -4.5 | 5.39E-16 | 5.5 | 1.69E-29 | Type VI secretion system tube protein Hcp |
| W909_RS06255 | -3.3 | 3.79E-45 | 3.3 | 1.20E-39 | Type VI secretion system tip protein VgrG |
| W909_RS06380 | 1.8 | 0.00073064 | -2.1 | 9.64E-05 | Type VI secretion system-associated FHA domain protein TagH |
| W909_RS12540 | -3.7 | 1.37E-87 | 3.4 | 1.35E-66 | Type VI secretion system tip protein VgrG |
| W909_RS12545 | -5 | 5.59E-34 | 5.6 | 3.29E-47 | Type VI secretion system tube protein Hcp |
| **Acyltransferase** |  |  |  |  |  |
| W909_RS00965 | 2.2 | 2.47E-10 | -2.1 | 1.95E-10 | Acetyl-CoA C-acyltransferase FadA |
| W909_RS11730 | -2.7 | 6.21E-108 | 2.8 | 5.60E-112 | Phosphate acyltransferase PlsX |
| W909_RS13030 | 2.3 | 4.48E-45 | -2.3 | 6.47E-47 | Phosphate acetyltransferase |
| **Others** |  |  |  |  |  |
| W909_RS04875 | -2.3 | 4.84E-40 | 3.2 | 2.25E-89 | Polyamine aminopropyltransferase |
| W909_RS13025 | 2.4 | 5.84E-154 | -2.6 | 6.62E-177 | Acetate kinase |
| W909_RS00225 | -1.9 | 0.00039944 | 2.5 | 2.97E-06 | Lipid kinase |
| W909_RS00950 | -1.8 | 6.37E-07 | 2 | 1.16E-07 | Transcription/translation regulatory transformer protein RfaH |
| W909_RS01290 | 2.4 | 1.27E-31 | -2.4 | 2.55E-31 | SDR family oxidoreductase |
| W909_RS01300 | -2.5 | 1.97E-203 | 2.9 | 6.88E-278 | tRNA dihydrouridine synthase DusB |
| W909_RS01450 | 3.2 | 3.88E-105 | -3.3 | 9.63E-116 | Ribosome hibernation promoting factor |
| W909_RS02340 | 2.4 | 9.48E-11 | -2.4 | 7.80E-11 | Chorismate mutase |
| W909_RS02520 | -2.1 | 9.59E-17 | 2.3 | 3.55E-20 | Molecular chaperone OsmY |
| W909_RS02985 | -2.2 | 6.48E-165 | 2.4 | 5.03E-186 | Preprotein translocase subunit SecG |
| W909_RS03000 | -2 | 7.05E-92 | 2.2 | 3.25E-114 | Ribosome maturation factor RimP |
| W909_RS04090 | 1.7 | 2.82E-194 | -2 | 1.44E-273 | NAD(P)H-binding protein |
| W909_RS04265 | 2.9 | 0 | -2.2 | 1.48E-217 | Catalase |
| W909_RS04570 | -2.1 | 1.08E-05 | 2.1 | 3.90E-05 | YgdI/YgdR family lipoprotein |
| W909_RS04685 | -2.3 | 0.00014746 | 2.1 | 0.0010016 | YgdI/YgdR family lipoprotein |
| W909_RS04755 | -3 | 0 | 2.9 | 0 | tRNA (guanosine(37)-N1)-methyltransferase TrmD |
| Gene ID | Fold change  (∆*speA*/∆*speA*+Put) | Pval | Fold change  (EC1/∆*speA*) | Pval | Functiom |
| W909_RS04785 | 2.6 | 0 | -2.6 | 0 | Ribosomal subunit interface protein |
| W909_RS05100 | -3.2 | 1.32E-29 | 3.9 | 2.74E-49 | Nitrogen regulatory protein P-II 2 |
| W909_RS05115 | -2.2 | 4.99E-66 | 2 | 5.72E-45 | Glycoprotein/polysaccharide metabolism |
| W909_RS05550 | -2.1 | 2.33E-21 | 2.4 | 2.41E-28 | 23S rRNA (pseudouridine(1915)-N(3))-methyltransferase RlmH |
| W909_RS05555 | -2 | 4.76E-39 | 2.3 | 1.36E-50 | Ribosome silencing factor RsfS |
| W909_RS06075 | -2.4 | 4.24E-10 | 2.2 | 3.74E-08 | Polysaccharide export protein Wza |
| W909_RS06150 | -2.5 | 2.06E-09 | 2 | 2.74E-05 | Mannose-1-phosphate guanylyltransferase |
| W909_RS07170 | 4 | 7.23E-45 | -3.9 | 2.98E-44 | Diaminobutyrate--2-oxoglutarate transaminase |
| W909_RS07955 | -2.6 | 1.17E-21 | 3.3 | 7.45E-38 | Elongation factor P-like protein YeiP |
| W909_RS07960 | -2.2 | 0.0004057 | 2.7 | 9.96E-06 | Proteinase inhibitor |
| W909_RS08240 | -3.5 | 2.20E-138 | 3.7 | 7.17E-157 | Translation initiation factor IF-1 |
| W909_RS08385 | 1.9 | 1.02E-36 | -2.4 | 3.80E-54 | Bifunctional phosphoribosyl-AMP cyclohydrolase |
| W909_RS08390 | 1.9 | 3.62E-37 | -2.5 | 2.18E-57 | Imidazole glycerol phosphate synthase subunit HisF |
| W909_RS08395 | 2 | 2.32E-39 | -2.7 | 9.91E-61 | 1-(5-phosphoribosyl)-5-[(5-phosphoribosylamino)methylideneamino]imidazole-4-carboxamide isomerase |
| W909_RS08400 | 2.1 | 1.24E-30 | -2.7 | 1.26E-44 | Imidazole glycerol phosphate synthase subunit HisH |
| W909_RS08405 | 1.9 | 8.41E-54 | -2.5 | 6.95E-86 | Bifunctional histidinol-phosphatase |
| W909_RS08410 | 2.1 | 5.47E-83 | -2.4 | 1.05E-102 | Histidinol-phosphate transaminase |
| W909_RS08415 | 1.7 | 2.93E-55 | -2 | 3.82E-73 | Histidinol dehydrogenase |
| W909_RS09055 | -2.6 | 9.12E-50 | 3 | 1.41E-71 | Trimeric intracellular cation channel family protein |
| W909_RS09455 | 2 | 3.01E-17 | 2 | 2.08E-18 | Serine 3-dehydrogenase |
| W909_RS09460 | 4.1 | 1.32E-174 | -3.2 | 1.75E-145 | Serine 3-dehydrogenase |
| W909_RS09490 | 2.6 | 0 | -2.6 | 0 | Serine 3-dehydrogenase |
| W909_RS09690 | 2.6 | 3.27E-147 | -2.2 | 3.28E-121 | Xylanase |
| W909_RS09870 | -2.1 | 6.76E-54 | 2.6 | 4.00E-95 | Membrane protein |
| W909_RS09980 | 1.7 | 1.74E-31 | -2.2 | 8.91E-49 | Integration host factor subunit alpha |
| W909_RS10430 | 2.4 | 7.69E-09 | -3.2 | 2.12E-12 | HPr kinase |
| W909_RS10680 | -2.5 | 9.12E-40 | 2.2 | 1.47E-26 | Acyltransferase |
| W909_RS11210 | -5 | 4.27E-176 | 4.5 | 8.47E-114 | Osmotically-inducible lipoprotein OsmB |
| W909_RS11270 | -2.6 | 1.10E-29 | 2.6 | 1.07E-27 | Membrane-bound lysozyme inhibitor of C-type lysozyme |
| W909_RS11450 | -2.2 | 8.66E-22 | 2.4 | 2.79E-23 | Quinone-dependent dihydroorotate dehydrogenase |
| W909_RS11630 | -3.8 | 0 | 3.2 | 2.36E-190 | Glycine zipper 2TM domain-containing protein |
| W909_RS11740 | -2.7 | 0 | 2.8 | 0 | 23S rRNA accumulation protein YceD |
| W909_RS11865 | 2.5 | 1.08E-55 | -2.9 | 8.62E-69 | Phosphate starvation protein PhoH |
| W909_RS12620 | -2.3 | 0.00064096 | 2 | 0.0045394 | conjugal transfer protein TrbL |
| W909_RS13275 | -2.2 | 7.84E-59 | 2.8 | 4.37E-107 | Long-chain fatty acid transporter FadL |
| W909_RS13480 | -2.1 | 6.79E-10 | 2.1 | 8.55E-09 | IS3 family transposase |
| W909_RS14080 | 2 | 5.60E-13 | -2.6 | 1.10E-18 | Nuclear transport factor 2 family protein |
| W909_RS14085 | 2.1 | 4.66E-15 | -2.6 | 9.18E-21 | Short-chain dehydrogenase |
| W909_RS14090 | 2.6 | 3.46E-43 | -2.7 | 4.97E-46 | FAD-dependent oxidoreductase |
| W909_RS14100 | 2.7 | 2.53E-24 | -2.8 | 1.27E-25 | Class I SAM-dependent methyltransferase |
| Gene ID | Fold change  (∆*speA*/∆*speA*+Put) | Pval | Fold change  (EC1/∆*speA*) | Pval | Functiom |
| W909_RS14355 | -3.3 | 2.18E-29 | 3.8 | 2.45E-42 | Inositol-1-monophosphatase |
| W909_RS14455 | -2.4 | 2.31E-37 | 2.4 | 2.91E-33 | Membrane-bound lytic murein transglycosylase MltF |
| W909_RS14810 | -1.8 | 7.30E-29 | 2 | 2.03E-33 | tRNA preQ1(34) S-adenosylmethionine ribosyltransferase-isomerase |
| W909_RS15465 | -2.4 | 1.11E-08 | 2.9 | 2.53E-13 | 6-carboxytetrahydropterin synthase QueD |
| W909_RS15745 | -3.2 | 2.53E-73 | 3.6 | 1.41E-98 | Primosomal replication protein N |
| W909_RS15860 | 2.6 | 1.25E-36 | -2.1 | 6.43E-28 | 6-phospho-beta-glucosidase |
| W909_RS16040 | -2.6 | 1.04E-15 | 3.1 | 3.43E-23 | YitT family protein |
| W909_RS16525 | 3.8 | 2.86E-142 | -3.2 | 2.51E-125 | Tryptophanase |
| W909_RS16555 | 1.9 | 0 | -2.7 | 0 | Aacetolactate synthase |
| W909_RS16560 | 2.3 | 1.53E-72 | -2.5 | 1.03E-85 | Acetolactate synthase isozyme 1 small subunit |
| W909_RS16670 | -1.9 | 3.90E-21 | 2.2 | 6.93E-27 | LysE family translocator |
| W909_RS16680 | -2.8 | 9.84E-23 | 3.2 | 1.47E-30 | Carbamoyl-phosphate synthase small subunit |
| W909_RS16775 | 1.7 | 7.65E-84 | -2.4 | 7.93E-158 | Threonine synthase |
| W909_RS16780 | 1.4 | 2.38E-30 | -2.2 | 4.02E-67 | Homoserine kinase |
| W909_RS16785 | 1.6 | 8.36E-145 | -2.3 | 8.17E-275 | Bifunctional aspartate kinase/homoserine dehydrogenase I |
| W909_RS17185 | 1.7 | 1.52E-21 | -2 | 2.16E-30 | [acyl-carrier-protein] S-malonyltransferase |
| W909_RS17190 | 2.1 | 1.31E-43 | -2.1 | 8.43E-46 | [acyl-carrier-protein] S-malonyltransferase |
| W909_RS17205 | 3.2 | 0 | -2.9 | 0 | Polyketide synthase |
| W909_RS17210 | 3.1 | 3.53E-239 | -3 | 6.14E-237 | 6-deoxyerythronolide-B synthase |
| W909_RS17225 | 4.6 | 2.11E-39 | -4.5 | 3.40E-39 | Acyl carrier protein |
| W909_RS17230 | 4 | 0 | -3.4 | 0 | Beta-ketoacyl-[acyl-carrier-protein] synthase II |
| W909_RS17235 | 4.7 | 6.68E-135 | -3.8 | 5.20E-119 | LLM class flavin-dependent oxidoreductase |
| W909_RS17240 | 4.5 | 0 | -4 | 0 | 6-methylsalicylic acid synthase |
| W909_RS17245 | 4.4 | 0 | -3.7 | 9.46E-290 | Monooxygenase |
| W909_RS17250 | 3.8 | 0 | -3.7 | 0 | Polyketide synthase |
| W909_RS17260 | 3.2 | 1.73E-301 | -2.9 | 2.23E-273 | Poly(3-hydroxyalkanoate) depolymerase |
| W909_RS17265 | 3.7 | 0 | -3.2 | 0 | 3-hydroxy-3-methylglutaryl-ACP synthase |
| W909_RS17270 | 2.9 | 4.75E-78 | -2.7 | 1.10E-75 | Enoyl-CoA hydratase |
| W909_RS17275 | 3.3 | 1.90E-89 | -2.8 | 5.79E-78 | Enoyl-CoA hydratase |
| W909_RS18120 | 2.8 | 1.68E-103 | -2.3 | 1.89E-83 | Glycogen phosphorylase |
| W909_RS18125 | 2.6 | 1.64E-30 | -2.4 | 1.25E-27 | Glycogen synthase GlgA |
| W909_RS18150 | -3.5 | 0 | 3.1 | 0 | Cold-shock protein |
| W909_RS18535 | 1.8 | 9.48E-86 | -2.1 | 2.07E-115 | Branched-chain-amino-acid transaminase |
| W909_RS18545 | 2 | 6.75E-89 | -3.2 | 4.81E-172 | Acetolactate synthase 2 catalytic subunit |
| W909_RS19335 | -2.3 | 1.49E-28 | 2.5 | 3.48E-34 | Long-chain-fatty-acid--CoA ligase |
| W909_RS19340 | -1.9 | 2.06E-10 | 3 | 4.20E-29 | Short chain dehydrogenase |
| W909_RS19415 | -2.5 | 0 | 2.9 | 0 | Glutamate--ammonia ligase |
| W909_RS19420 | -2.1 | 1.33E-26 | 3.1 | 1.26E-70 | Nitrogen regulation protein NR(II) |
| W909_RS19545 | -1.9 | 4.49E-37 | 2 | 2.41E-38 | FMN-binding protein MioC |
| W909_RS19560 | -1.6 | 1.31E-79 | 2.1 | 3.87E-139 | F0F1 ATP synthase subunit I |
| W909_RS19730 | 2.2 | 2.52E-48 | -2.3 | 1.54E-51 | 4-hydroxyphenylpyruvate dioxygenase |
| W909_RS19755 | -2.1 | 7.42E-08 | 2.2 | 5.03E-08 | Rribonuclease P protein component |
| Gene ID | Fold change  (∆*speA*/∆*speA*+Put) | Pval | Fold change  (EC1/∆*speA*) | Pval | Functiom |
| W909_RS19810 | 2.1 | 3.56E-92 | -2.6 | 2.37E-133 | Non-ribosomal peptide synthetase |
| W909_RS20220 | 2.2 | 0 | -2.2 | 0 | Ribosome modulation factor |

^a^ EC1, ∆*speA* and ∆*speA*+Put indicated the wild type strain of *Dickeya zeae*, *speA* gene deletion mutant and the mutant of ∆*speA* cultured in swimming motility medium with putrescine at final concentration of 0.1 mM, respectively.

**Supplementary Figure S1**

**
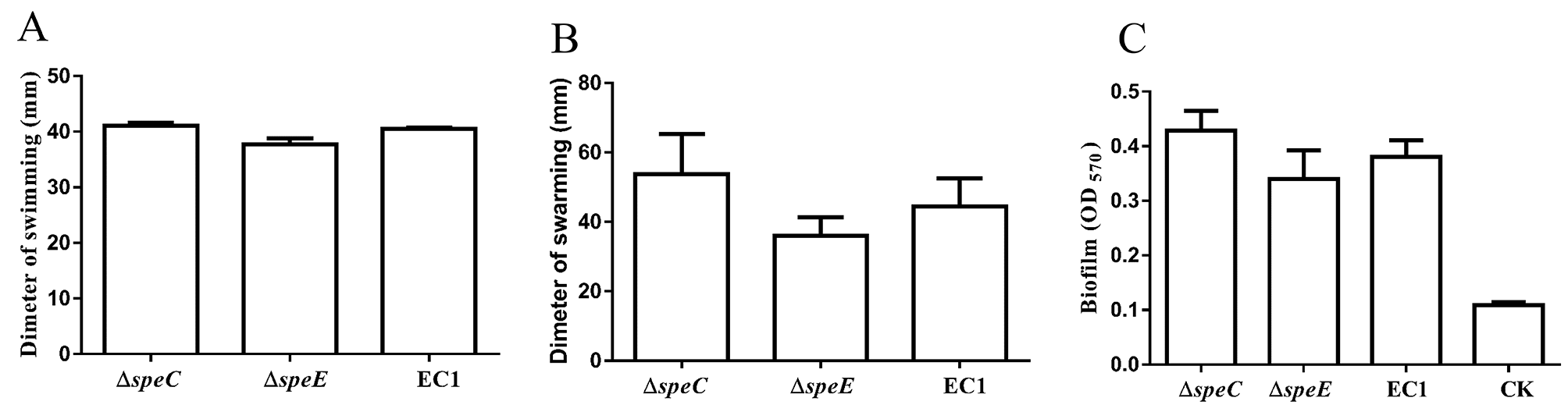
**

**Supplementary Figure S1.** Effects of disruption enzymes of polyamine biosynthesis on cell motility and biofilm formation. (A) swimming motility, (B) swarming motility and (C) biofilm formation. EC1, ∆*speC* and ∆*speE* indicated the wild type strain, *speC* gene deletion mutant and *speE* gene deletion mutant of *Dickeya zeae*, respectively. CK indicated SOBG medium.

**Supplementary Figure S2**

**

**

**Supplementary Figure S2.** Growth curves of *Dickeya zeae* EC1 and its derivatives in the minimal medium. ∆*speA* indicated the *speA*-deletion mutant and ∆*speA*∆*potF*∆*plaP* was the *potF*-*plaP*-deletion mutant in *speA*-deletion genetic background.

**Supplementary Figure S3**

**
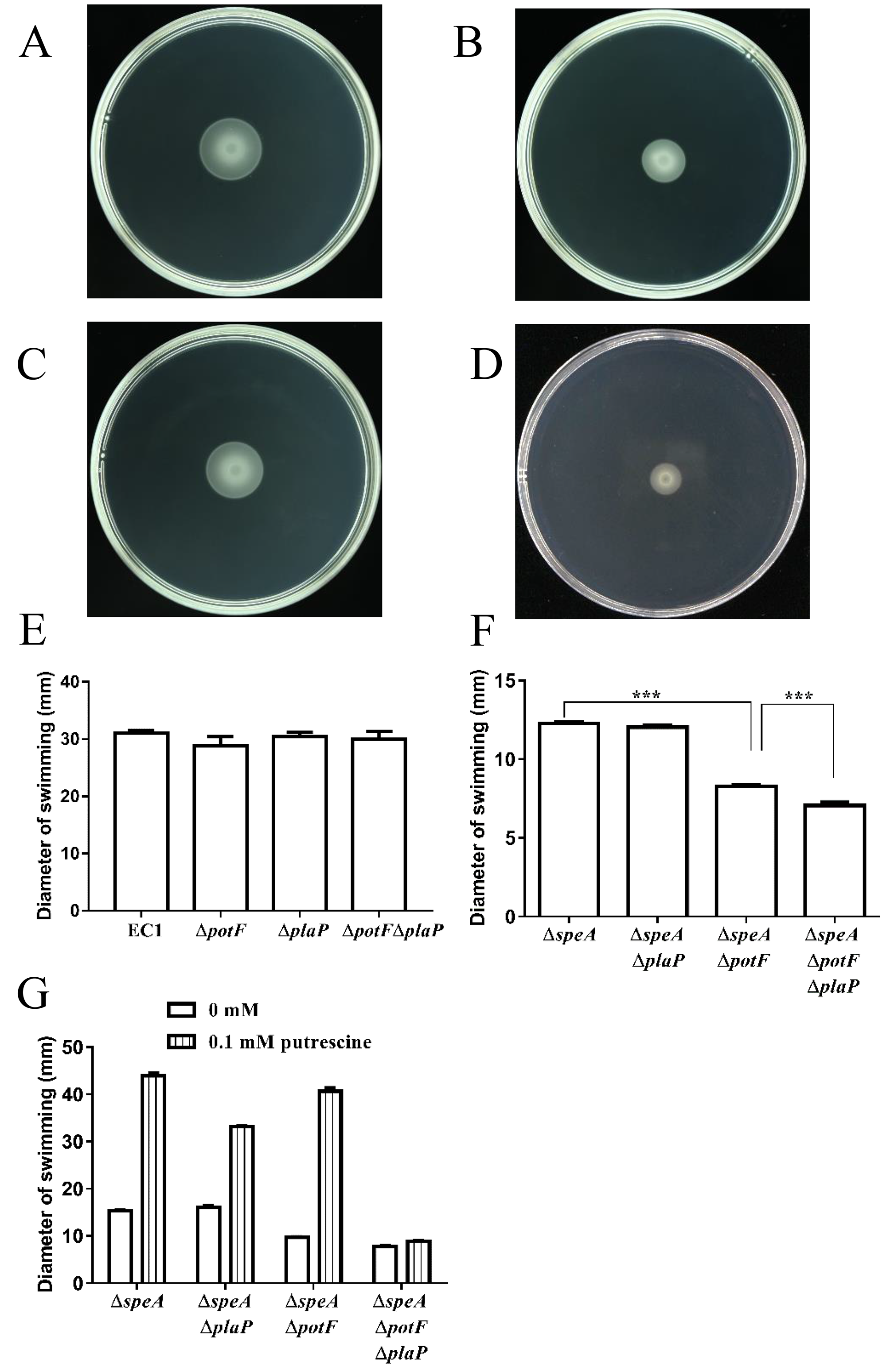
**

**Supplementary Figure S3.** Influence of putrescine transporters on swimming motility. (A) *speA*-disrupted mutant, (B) the *potF*-*plaP*-disrupted mutant in the *speA*-disrupted genetic background, the complemented strains (C) ∆*speA*∆*potF*∆*plaP*(pBBRI-*potF*) and ∆*speA*∆*potF*∆*plaP*(pBBRI-*plaP*) (D), (E) and (F) Measurement of swimming motility of EC1 and its derivatives, (G) Measurement of swimming motility of mutant ∆*speA* and its derivatives with or without putrescine. ∆*speA*, ∆*speA*∆*plaP*, ∆*speA*∆*potF* and ∆*speA*∆*potF*∆*plaP* indicated the *speA*-deletion mutant of *Dickeya zeae*, *plaP*-deletion mutant in *speA*-deletion genetic background, *potF*-deletion mutant in *speA*-deletion genetic background and *plaP*-*potF*-deletion mutant in *speA*-deletion genetic background, respectively. The photographs were taken after incubation for 24 h at 28 ℃. Error bars denote standard errors. ***, corrected *P* value *potF*-of <0.001.

**Supplementary Figure S4**

**

**

**Supplementary Figure S4.** Effect of exogenous addition of polyamines on bacterial swimming motility. Bacterial strains were grown under the same condition but supplemented with putrescine (Put), spermidine (Spd), and spermine (Spe), respectively, at a final concentration of 0.1 mM. ∆*speA* indicated the *speA*-deletion mutant and ∆*speA*∆*potF*∆*plaP* was the *potF*-*plaP*-deletion mutant in *speA*-deletion genetic background. The diameters of swimming motility were measured after incubation for 24 h at 28 ℃. The data shown are the mean ± SE (n = 3).

**Supplementary Figure S5**

**
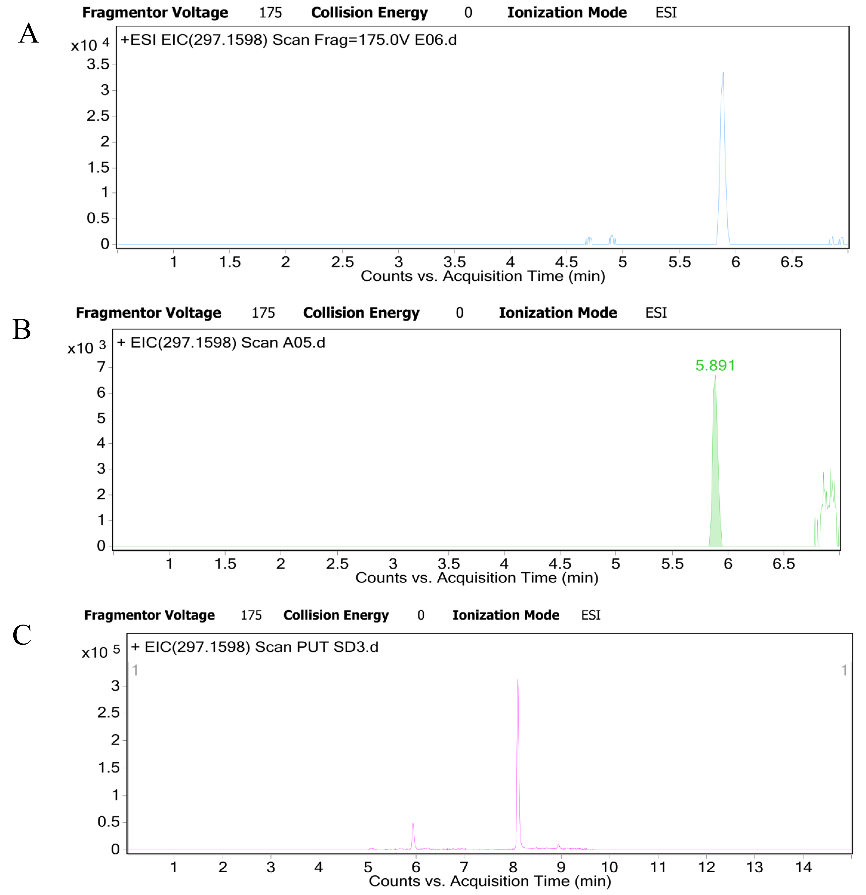
**

**Supplementary Figure S5.** High-performance liquid chromatography coupled with mass spectrometry (LC-MS) detection the concentration of benzoyled putrescine of *Dickeya zeae* EC1 and the *speA*-disrupted mutant ∆*speA* cells. (A) wild type, (B) mutant and (C) PUT (standard).

**Supplementary Figure S6**


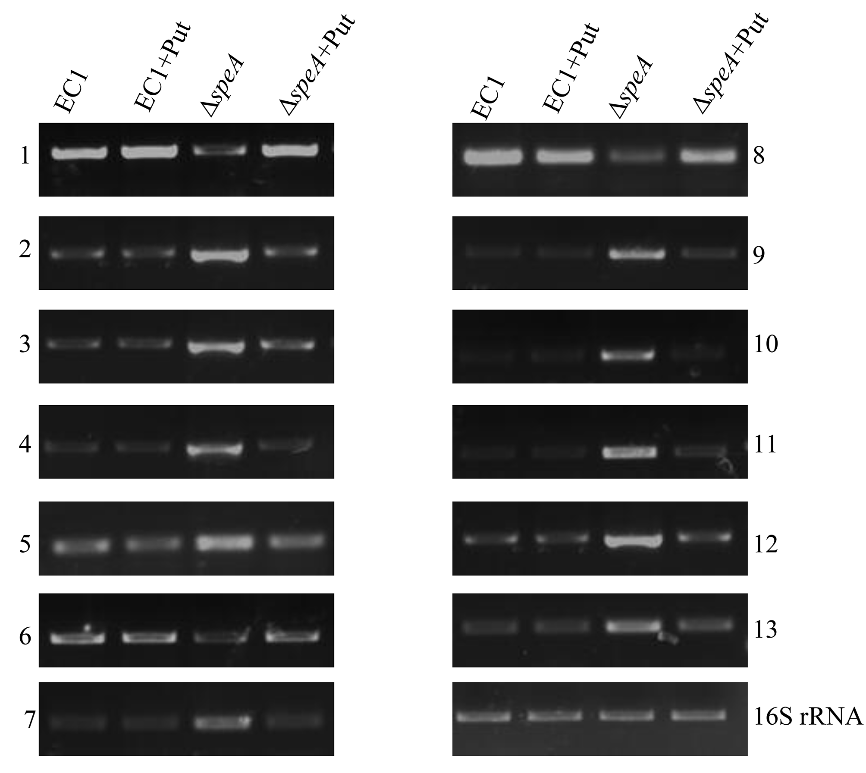


**Supplementary Figure S6.** RT-PCR analysis of putrescine signal on modulating genes expression. Gene ID of gene 1, 2, 3, 4, 5, 6, 7, 8, 9, 10, 11, 12 and 13 is W909_RS04875, W909_RS09730, W909_RS10380, W909_RS10395, W909_RS10435, W909_RS10550, W909_RS12085, W909_RS12490, W909_RS13025, W909_RS13030, W909_RS18920, W909_RS17140 and W909_RS12150, respectively. The reference gene of 16S rRNA was used standardizing the samples of RNA. EC1 and ∆*speA* indicated the wild type strain and *speA* gene deletion mutant of *Dickeya zeae*, respectively; EC1+Put and ∆*speA*+Put indicated the strains of EC1 and ∆*speA* cultured in swimming motility medium with putrescine at final concentration of 0.1 mM, respectively.
